# Covalently Interlocked Electrode–Electrolyte Interface for High‐Energy‐Density Quasi‐Solid‐State Lithium‐Ion Batteries

**DOI:** 10.1002/advs.202417143

**Published:** 2025-04-16

**Authors:** Dong‐Yeob Han, Im Kyung Han, Jin Yong Kwon, Seoha Nam, Saehyun Kim, Youngjin Song, Yeongseok Kim, Youn Soo Kim, Soojin Park, Jaegeon Ryu

**Affiliations:** ^1^ Department of Chemistry and Department of Battery Engineering Pohang University of Science and Technology (POSTECH) Pohang 37673 Republic of Korea; ^2^ Department of Materials Science and Engineering Pohang University of Science and Technology (POSTECH) Pohang 37673 Republic of Korea; ^3^ Department of Chemical and Biomolecular Engineering Sogang University Seoul 04107 Republic of Korea

**Keywords:** electrode–electrolyte interface, high‐energy‐density lithium‐ion batteries, interlocking system, quasi‐solid‐state batteries, silicon microparticle anodes

## Abstract

Quasi‐solid‐state batteries (QSSBs) are attracting considerable interest as a promising approach to enhance battery safety and electrochemical performance. However, QSSBs utilizing high‐capacity active materials with substantial volume fluctuations, such as Si microparticle (SiMP) anodes and Ni‐rich cathodes (NCM811), suffer from unstable interfaces due to contact loss during cycling. Herein, an in situ interlocking electrode–electrolyte (IEE) system is introduced, leveraging covalent crosslinking between acrylate‐functionalized interlocking binders on active materials and crosslinkers within the quasi‐solid‐state electrolyte (QSSE) to establish a robust, interconnected network that maintains stable electrode–electrolyte contact. This IEE system addresses the limitations of liquid electrolyte and QSSE configurations, evidenced by low voltage hysteresis in (de)lithiation peaks over 200 cycles, stable interfacial resistance throughout cycling, and the absence of void formation. A pressure‐detecting cell kit further confirms that the IEE system exhibits lower pressure changes during cycling without any voltage fluctuations from contact loss. Moreover, the SiMP||NCM811 full cell with the IEE system demonstrates superior electrochemical performance, and a bi‐layer pouch cell configuration achieves an impressive energy density of 403.7 Wh kg^−1^/1300 Wh L^−1^, withstanding mechanical abuse tests such as folding and cutting, providing new insights into high‐energy‐density QSSBs.

## Graphical abstract

The interlocking electrode–electrolyte (IEE) system addresses the challenges of quasi‐solid‐state electrolyte systems, such as void formation and increased interfacial resistance, in silicon microparticle anodes and Ni‐rich layered cathodes. By preserving a stable electrode–electrolyte interface, the IEE system mitigates mechanical degradation and delivers superior electrochemical performance, enabling high‐energy‐density quasi‐solid‐state batteries.

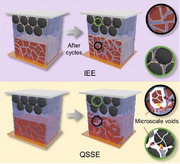

## 1 Introduction

The global shift toward electrification has catalyzed significant growth in markets such as electric vehicles, unmanned aerial vehicles, high‐performance electronics, and distributed energy storage systems.^[^ ^1^ ^]^ As a result, there is an increasing demand for advanced electrochemical energy storage systems that exceed the performance limits of conventional lithium‐ion batteries (LIBs). The energy density of traditional LIB configurations, featuring LiCoO_2_ or LiFePO_4_ cathodes paired with graphite anodes, has reached its theoretical limit.^[^ ^2^ ^]^ Integrating high‐capacity silicon (Si) anodes (3579 mAh g⁻¹ with a low operating potential of ≈0.2 V vs Li/Li⁺) and Ni‐rich layered cathodes, such as LiNi_0.8_Co_0.1_Mn_0.1_O_2_ (NCM811) (200 mAh g⁻¹ at 4.3 V vs Li/Li⁺), has emerged as a promising approach to significantly enhancing energy density in LIBs.^[^ ^3^ ^]^

However, the reliance on liquid electrolytes (LEs) in conventional LIBs poses safety risks and limits long‐term stability, particularly in high‐energy‐density configurations. Solid‐state electrolytes (SSEs) have, therefore, gained attention as a next‐generation solution, offering improved safety and higher energy density by replacing flammable LEs.^[^ ^4^ ^]^ Despite their promise, critical challenges remain, including poor electrode–electrolyte interface stability, sluggish kinetics, and high operating pressure.^[^ ^5^ ^]^ For Ni‐rich cathodes such as NCM811, mechanical and chemical degradation at the interface, exacerbated by volume changes during cycling, significantly limits performance.^[^ ^6^ ^]^ In particular, intragranular cracking of those cathodes triggers the formation of interfacial gaps between the active materials and the SSEs, resulting in increased interfacial resistance, degraded Li‐ion kinetics, and poor cycle performance, along with a low initial Coulombic efficiency (ICE).^[^ ^7^ ^]^ On the anode side, SSEs provide enhanced mechanical support toward the large‐volume‐change anodes, thereby promoting the use of Si microparticles (SiMPs) in solid‐state batteries (SSBs) rather than Si nanoparticles (SiNPs).^[^ ^5^, ^8^ ^]^ Given the increased rigidity of SSEs, carbon‐free SiMP anodes for SSBs were reported, which achieved long‐cycle performance at a high current density.^[^ ^9^ ^]^ However, the configuration of SiMP||NCM811 full cells exhibits only about half of their theoretical capacity due to interface instability and sluggish electrode kinetics. In particular, lithium extraction from the fully charged SiMP anodes results in contact loss and the formation of large voids between the SiMP anode and the SSE, which leads to increased interfacial resistance and failure of subsequent cycles.^[^ ^5^, ^10^ ^]^ Securing contact in SSBs typically requires high stack pressures, up to even several tens to hundreds of megapascals (MPa) during cell assembly and operation, which is unrealistic for practical application.^[^ ^11^ ^]^ Moreover, the operation of typical SSBs necessitates high‐temperature conditions due to the intrinsically low ionic conductivity of SSEs.^[^ ^12^ ^]^

Quasi‐solid‐state electrolytes (QSSEs), which encapsulate LEs within a polymer matrix, have been proposed and extensively studied owing to their structural flexibility and high compatibility with the differently shaped battery electrodes, thereby partly addressing the electrode–electrolyte contact issue.^[^ ^13^ ^]^ QSSEs also exhibit comparable ionic conductivity to LEs, enabling operation at room temperature while offering enhanced safety compared to conventional LEs. A supremely elastic QSSE has been developed to serve as an intraelectrode cushion to reduce the expansion of SiNPs during lithiation and to facilitate electrode recovery during delithiation.^[^ ^14^ ^]^ Additionally, fluorinated carbon‐incorporated SiMPs with elastic QSSE have demonstrated the ability to partially dissipate the stress associated with the volume expansion of SiMPs.^[^ ^15^ ^]^ However, even molecularly engineered elastic QSSEs are incapable of fully accommodating the substantial volume contraction of high‐capacity active materials, especially SiMPs, during delithiation as frequently complemented by auxiliary methods such as the restriction of particle size to submicron scales or partial utilization of Si capacity (e.g., state‐of‐charge (SOC) control) which ultimately reduce the overall energy density of the battery. To this end, the interlocking of active materials with the QSSE is essential for sustaining an intimate and stable electrode–electrolyte interface throughout battery cycling, thereby preventing the void formation and capacity loss.

**Scheme 1:**
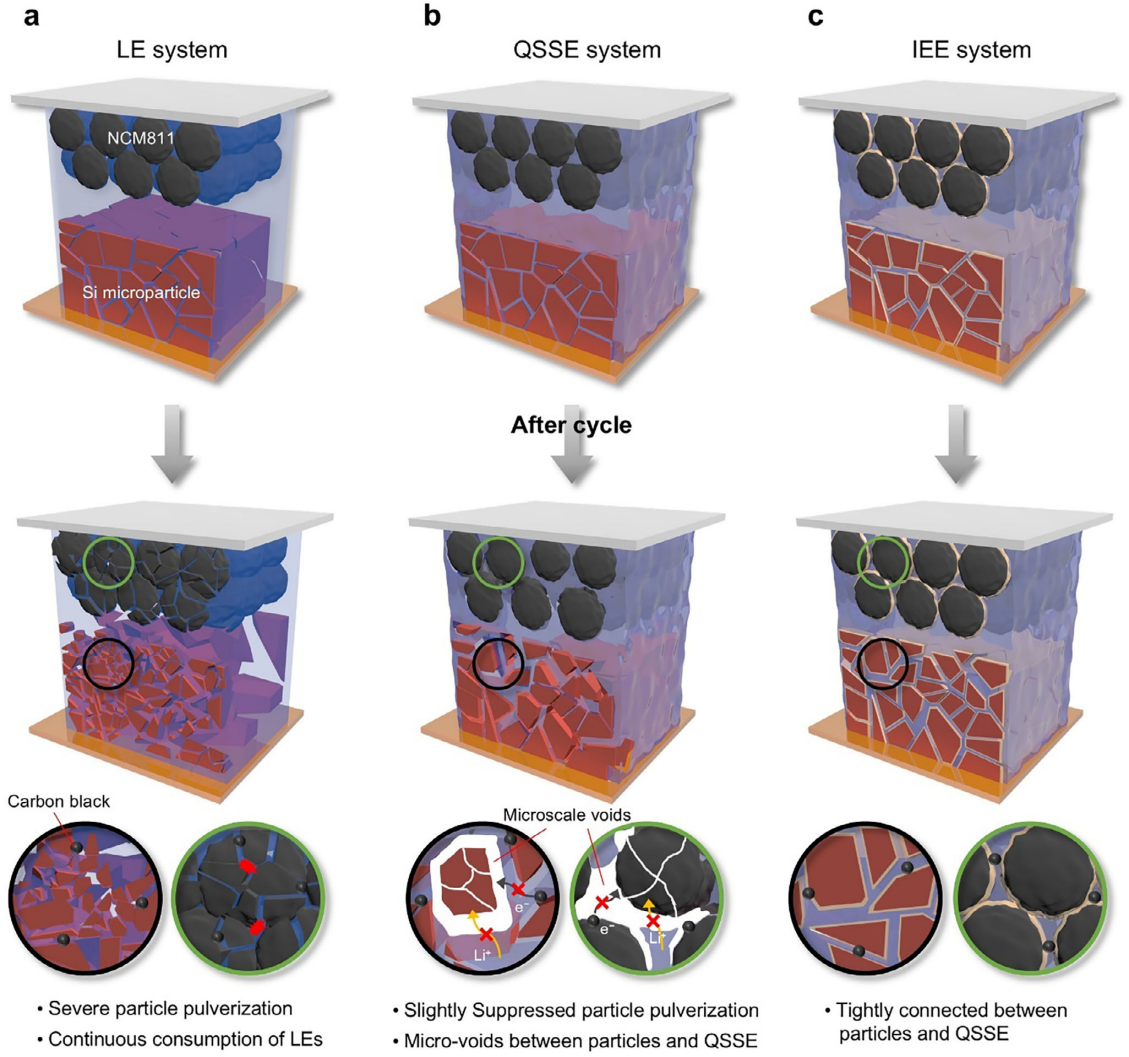
Schematic illustration of three different systems. Graphical depictions before and after the cycle along with challenges from a) LE system, b) QSSE system, and c) IEE system.

Herein, an in situ interlocking electrode–electrolyte (IEE) system for monolithic full cells is proposed, enabling the stable operation of SiMP anodes and Ni‐rich layered cathodes in quasi‐solid‐state batteries (QSSBs). The IEE is realized through covalent crosslinking between acrylate groups on a designed interlocking binder (IB) on the active materials and those on a crosslinker during QSSE formation. This interconnected network ensures stable and intimate contact between the electrode and electrolyte throughout cycling, preventing capacity decay and reducing charge transfer resistance. In comparison, the LE system suffered from severe SiMP pulverization due to drastic volume fluctuations and continuous side reactions with NCM811, resulting in significant capacity decay within five cycles (**Scheme** **1**a). Although the QSSE system mitigated volume expansion during lithiation, it could not fully recover during delithiation, forming substantial amounts of microscale voids between the active materials and the QSSE formed at the electrode–electrolyte interface (Scheme 1b). Consequently, interfacial resistance sharply increased, and further utilization of isolated active materials was prohibited. By tightly interlocking the active materials and QSSE, the IEE system maintained an intimate electrode–electrolyte interface and mitigated mechanical degradation, delivering superior electrochemical performance with low interfacial resistance (Scheme 1c). Specifically, the SiMP half cell with the IEE system demonstrated 81.3% capacity retention after 200 cycles, with full utilization of the Si capacity (SOC ≈ 100%). Furthermore, the SiMP||NCM811 monolithic full cell exhibited stable cycling behavior, with quick Coulombic efficiency (CE) stabilization at 99.5% within three cycles. Additionally, a bi‐layer pouch‐type monolithic full cell with the IEE system achieved a high‐energy density of 403.7 Wh kg^−1^/1300 Wh L^−1^, surpassing previously reported Si‐based full cells. The IEE pouch cell also demonstrated enhanced safety, showing stable operation under mechanical abuse, such as folding and cutting, confirming its feasibility for high‐energy‐density applications.

## 2 Results and Discussion

**Figure 1:**
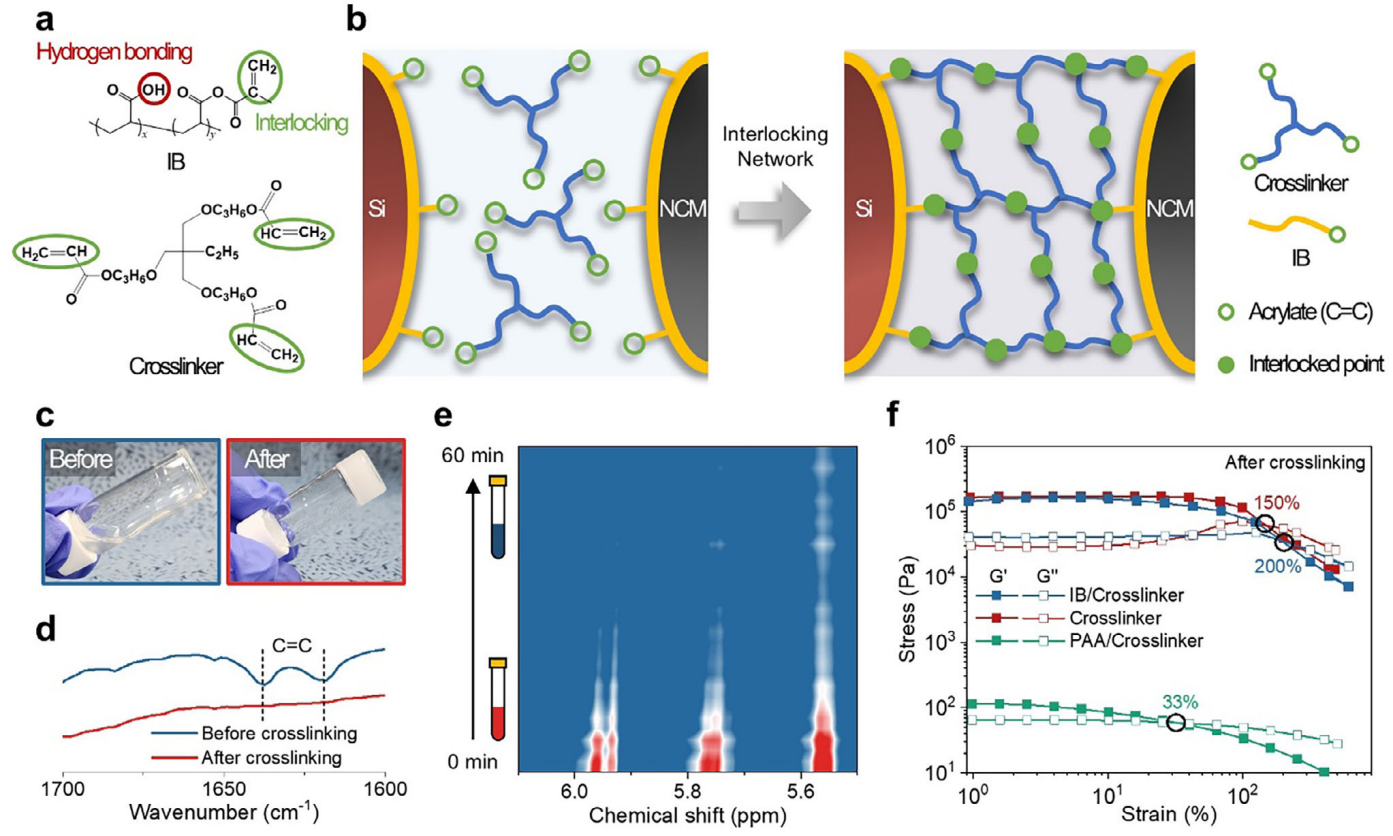
Characterization of the IEE system. a) Molecular structures of IB and crosslinker used in the IEE system. b) Schematic illustration of the interlocking process between electrodes and electrolytes. c) Digital photos and d) FT‐IR spectra of a quasi‐solid gel with IB and crosslinker before and after crosslinking. e) In real‐time monitoring of forming a quasi‐solid gel with IB and crosslinker at 60 °C using in situ ^1^H NMR. f) Strain amplitude sweep of different quasi‐solid gels at a frequency of 1 rad s^−1^.

The IB was designed and synthesized to perform both binding and interlocking functions via a simple grafting reaction of polyacrylic acid (PAA) with methacrylic anhydride (MA) in phosphate‐buffered saline (PBS, pH = 7.4) at 50 °C (Figure S1a, Supporting Information).^[^ ^16^ ^]^ Methacrylate groups (interlocking sites) were introduced on the carboxyl (−COOH) groups of PAA, providing interlocking sites capable of crosslinking under thermal treatment. The ^1^H nuclear magnetic resonance (NMR) spectroscopy confirmed successful substitution, revealing that ≈33% of the hydroxyl groups were substituted with methacrylate groups (Figure S1, Supporting Information). Importantly, the remaining carboxyl groups enabled hydrogen bonding with the high‐capacity active materials (Si and NCM particles) and current collectors (Cu and Al foils), thereby improving interfacial adhesion and stability (**Figure** **1**a). This hydrogen bonding arises from the surface oxide layers present on Si and NCM811, which contain oxygen‐rich functional groups capable of interacting with carboxyl groups in IB.^[^ ^3^, ^17^ ^]^ Similarly, Cu and Al current collectors develop native oxide layers upon air exposure, facilitating additional hydrogen bonding with IB.^[^ ^18^ ^]^ This dual‐functionality allowed the IB to act as an “*interlocker*” on the electrode side through acrylate groups, while trimethylolpropane propoxylate triacrylate (TMPTA) served as a crosslinker for the electrolyte side. This resulted in a robust IEE system through the in situ polymerization of TMPTA, tightly bonding the IB to the electrolyte during thermal curing (Figure 1b).

To validate the interlocking network, a model system was developed with IB (or PAA for comparison), TMPTA, benzoyl peroxide (initiator), and a cosolvent. The component weight ratio was 1:6:0.2:93, with the solvent comprising distilled water and an organic solvent in a 2:8 ratio. A cosolvent was necessary due to the differential solubility of IB and TMPTA; IB dissolves only in water, while TMPTA dissolves in organic solvents. Heating the precursor solution at 60 °C for 1 h resulted in a quasi‐solid gel (QSG) via in situ polymerization of the crosslinker (Figure 1c). Fourier‐transform infrared (FT‐IR) spectroscopy showed the disappearance of C═C characteristic stretching vibrations at 1618 and 1637 cm^−1^, confirming the crosslinking reaction of acrylate groups in IB and TMPTA (Figure 1d).^[^ ^19^ ^]^ In situ ^1^H NMR monitored real‐time crosslinking dynamics with a similar model system prepared using deuterated water (D_2_O). The initial ^1^H NMR spectra showed distinct vinyl proton peaks at 5.95, 5.76, and 5.56 ppm (Figure 1e).^[^ ^20^ ^]^ As the system was heated at 60 °C, these peaks gradually diminished with near complete disappearance after 1 h, signifying the completion of crosslinking. Combined FT‐IR and in situ ^1^H NMR analysis confirmed successful interlocking between IB and TMPTA, forming a robust network at 60 °C within 1 h.

Differential scanning calorimetry analysis of QSGs before crosslinking showed a *T_g_* of 119.7 °C. Upon crosslinking, the formation of a crosslinked network restricts chain mobility, resulting in an increased *T_g_* (*T_g1_* = 177.7 °C, *T_g2_* = 194.6 °C), shifting it to a higher temperature region. (Figure S2, Supporting Information). Additionally, the mechanical properties of QSGs with different configurations were assessed via rheological tests. A strain amplitude sweep from 1% to 500% at a constant frequency of 1 rad s^−1^ provided modulus measurements (Figure 1f). The gels displayed higher storage modulus (*G*′) than loss modulus (*G*″) at lower strains, typical solid‐like behavior, with *G*′ and *G*″ intersectioning at higher strain values, marking the transition from gel to sol behavior.^[^ ^21^ ^]^ Notably, the IB/crosslinker gel showed the highest cross‐over strain of 200%, highlighting its superior mechanical integrity. Compared to the gel with only a crosslinker (≈150% cross‐over strain), the enhanced properties of IB/crosslinker are attributed to effective crosslinking between the long polymer chains of IB and the poly(TMPTA) network, which dissipates mechanical stress efficiently.^[^ ^22^ ^]^ This interlocked network yields a cohesive structure with greater resilience and deformation tolerance.^[^ ^23^ ^]^ In contrast, the gel formed with PAA and the crosslinker (PAA/Crosslinker in Figure 1f) exhibited a much lower cross‐over strain (≈33%) and ≈200 times lower stress values. This is attributed to the lack of acrylate functional groups in PAA, preventing covalent crosslinking with TMPTA. Instead, physical entanglements between PAA chains interfere with effective crosslinking, resulting in a weaker network with inferior mechanical integrity. These findings demonstrate that interlocking between IB and TMPTA significantly enhances the structural integrity and robustness of QSG under mechanical stress.

To further evaluate the impact of covalent interlocking on interface adhesion, 180° peel‐off and shear stress tests were conducted on electrodes prepared with QSSE and IEE (Figure S3, Supporting Information). The IEE system exhibited significantly stronger adhesion than QSSE in both tests, with the shear adhesion force exceeding 2.2 kgf cm^−1^, more than twice that of QSSE. This improvement is attributed to the covalent bonding between IB and QSSE, which reinforces the electrode–electrolyte interface and prevents delamination. In contrast, QSSE displayed weaker adhesion, likely due to insufficient bonding at the interface. Strong interfacial adhesion in the IEE system is crucial for maintaining stable contact, reducing interfacial resistance, and mitigating mechanical degradation during prolonged cycling.

To assess the fundamental electrochemical performances of LE and QSSE, room‐temperature ionic conductivity, lithium transference number, and oxidative stability were measured. LE exhibited a slightly higher ionic conductivity of 9.13 × 10^−4^ S cm^−1^ compared to 7.63 × 10^−4^ S cm^−1^ for QSSE, likely due to the unrestricted ion mobility in LE, whereas the polymer matrix in QSSE introduces mild segmental restrictions (Figure S4a,b, Supporting Information). However, QSSE demonstrated a significantly higher lithium transference number (t_Li+_ = 0.61), indicating selective Li‐ion transport due to reduced anion mobility in the polymeric network (Figure S5, Supporting Information). Moreover, QSSE exhibited superior anti‐oxidative ability, maintaining a stable current response up to 6 V, while LE showed a slight current increase from 5 V, suggesting solvent decomposition at high voltage. This result confirms that QSSE possesses a wider electrochemically stable window, which is advantageous for high‐voltage battery applications (Figure S6, Supporting Information). To further examine whether the covalently crosslinked network in the IEE system affects ionic conductivity, we measured the ionic conductivity of PAA+QSSE (QSSE system) and IB+QSSE (IEE system) under identical conditions. Both systems exhibited nearly identical ionic conductivities (4.10 × 10^−5^ S cm^−1^ for the QSSE system and 4.09 × 10^−5^ S cm^−1^ for the IEE system), confirming that the IEE system does not compromise ionic conductivity (Figure S4c,d, Supporting Information). These results confirm that while QSSE has slightly lower ionic conductivity than LE, its improved transference number and oxidative stability enhance its suitability for high‐energy‐density applications.

**Figure 2:**
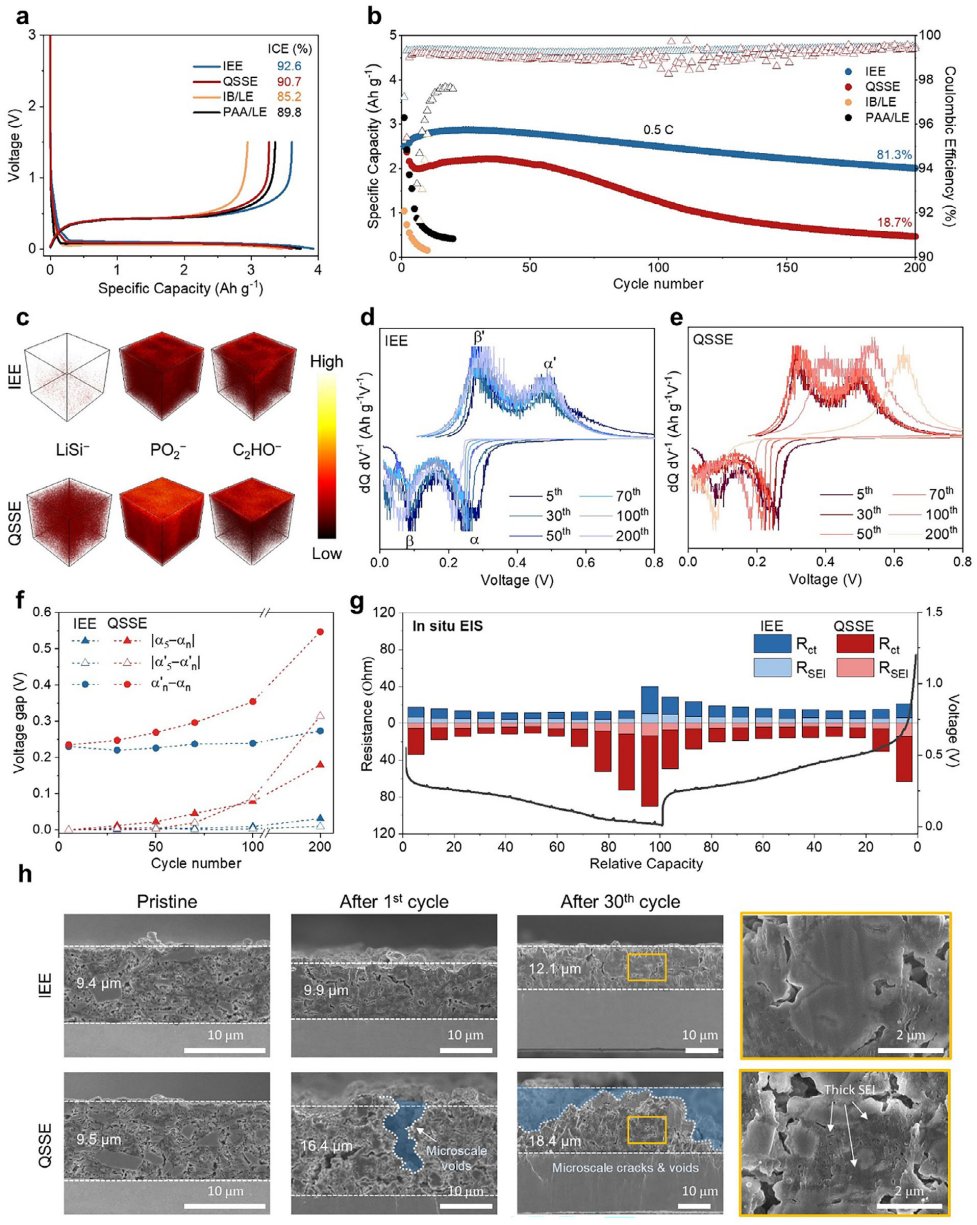
Electrochemical performance and stability of IEE system in SiMP half cells. a) Galvanostatic charge–discharge profiles of SiMP half cells with different systems. b) Long‐term cycling performance and corresponding CEs of Si electrode half cells in different systems at 0.5C (1C = 3.2 A g^−1^). c) TOF‐SIMS 3D reconstruction images for LiSi^−^, PO_2_^−^, and C_2_HO ^−^. Differential capacity curves (dQ/dV versus V) of SiMP half cells in d) IEE and e) QSSE systems. f) Voltage gaps between the lithiation and delithiation peaks in differential capacity curves across the cycles. g) In situ galvanostatic EIS measurements of Si electrodes with different systems (in situ characterization). h) Cross‐sectional SEM images of Si electrodes in the pristine state, after the 1^st^ cycle, and after the 30^th^ cycle with different systems.

The electrochemical performance of the IEE system was evaluated using coin‐type half cells, with SiMP as the working electrode and Li metal serving as both counter and (pseudo−)reference electrodes, respectively. The SiMPs used, with an average size of 3.0 µm, are relevant for industrial applications (Figure S7, Supporting Information). Four different systems were evaluated for their electrochemical behavior: IEE, QSSE, IB/LE, and PAA/LE. Both the IEE and QSSE systems employed the same electrolyte (6 wt.% TMPTA and 94 wt.% LE with 0.15 wt.% benzoyl peroxide) but different binders (IB for IEE and PAA for QSSE). IB/LE and PAA/LE systems lacked polymeric matrices, relying solely on LE and their respective binders. During the formation cycle at 0.05C (1C = 3.2 A g^−1^), the IEE system exhibited the highest ICE (>92%) and delivered near‐theoretical capacities (3606 mAh g^−1^), outperforming other systems (**Figure** **2**a; Table S1, Supporting Information). The superior performance of the IEE system is attributed to the tightly interlocked interface between SiMP electrodes and QSSE, enabling efficient Li‐ion transport and maintaining intimate electrode–electrolyte contact. While the QSSE system offered flexibility and buffering effects that enhanced initial ICE over PAA/LE, the absence of interlocking resulted in insufficient accommodation of SiMP volume changes, limiting performance. The IB/LE system showed the lowest ICE and capacity because, without crosslinking with the electrolyte, the acrylate groups in IB could not act as effective interlockers, and its lower hydroxyl group concentration limited its ability to accommodate initial SiMP expansion.

The long‐term cycling stability of SiMP half cells was evaluated at 0.5C up to 200 cycles (Figure 2b). The IB/LE and PAA/LE systems suffered from rapid capacity decay and low CEs, suggesting that polymeric binders alone cannot alleviate SiMP volume fluctuations. The QSSE system showed improved stability initially but exhibited gradual capacity and CE decay, retaining only 18.7% of its initial capacity after 200 cycles. This was attributed to the inability of QSSE to fully recover from SiMP contraction during delithiation, leading to microscale voids at the electrode–electrolyte interface that compromised Li‐ion transport. In contrast, the IEE system retained 81.3% of its initial capacity after 200 cycles. The tight interlocking in IEE prevented void formation, allowing consistent Li‐ion transport and minimizing mechanical degradation, with CE stabilizing above 99.5% within a few cycles. The IEE system also showed superior capacity retention at high current densities compared to the QSSE system, attributed to the stable interconnected network (Figure S8, Supporting Information). The versatility of the IEE system was further demonstrated by testing variations with different crosslinking monomers, including dipentaerythritol hexaacrylate (DPH) and pentaerythritol tetraacrylate (PTTA) (Figure S9, Supporting Information). The SiMP half cells with IEE using DPH and PTTA crosslinkers exhibited outstanding cycling performance, retaining 80.1% and 70.2% of their initial capacities after 200 cycles. This demonstrates that the interlocking mechanism ensures strong mechanical integrity and efficient Li‐ion transport, independent of the specific monomer used.

Insights into the interfacial stability of the IEE system were gained through SEI layer analysis, focusing on interfacial structures. Time‐of‐flight secondary ion mass spectrometry (TOF‐SIMS) revealed significant differences in SEI composition between QSSE and IEE systems (Figure 2c). QSSE showed higher PO_2_^−^ and C_2_HO^−^ intensities on SiMP surfaces, indicating SEI formed due to inhomogeneous parasitic reactions from contact loss. In contrast, the IEE system formed a thinner, more uniform SEI layer, as evidenced by lower PO_2_^−^ and C_2_HO^−^ intensities, reducing electrolyte decomposition and maintaining interfacial stability through its robust covalent network. Moreover, the QSSE system also showed higher LiSi^−^ signal intensity, indicating irreversible Li trapping, while the IEE system exhibited negligible residual Li, reflecting homogeneous and reversible lithiation/delithiation cycles, correlating with stable cycling performance and higher CE of the IEE system.^[^ ^24^ ^]^

The reversibility of electrochemical reactions was further assessed via differential capacity (dQ/dV) curves for SiMP half cells (Figure 2d,e). Both systems displayed broad lithiation (α and β) and delithiation (α′ and β′) peaks, corresponding to different Li*_x_* Si alloy phases.^[^ ^25^ ^]^ In the IEE system, lithiation peaks exhibited only slight shifts (less than 0.03 V) over 200 cycles, while delithation peaks remained unchanged, reflecting stable and reversible electrochemical processes. The stable peaks indicate consistent electrode–electrolyte contact in the IEE system, allowing repeatable lithiation/delithation of SiMPs with minimal capacity loss. In contrast, the QSSE system showed substantial peak shifts, where β and β′ became distorted and eventually disappeared, indicating interfacial degradation and increased resistance over time. The reversibility of the lithation/delithation processes was further quantified by calculating the voltage hysteresis between α and α′ peaks throughout cycling. For the IEE system, hysteresis remained relatively constant from the 5th to 100th cycle (<4% increase) and showed only a slight rise by the 200th cycle. The QSSE system, however, displayed a gradual increase in hysteresis starting from the 30th cycle, reaching 0.354 and 0.547 V by the 100th and 200th cycles, respectively. This corresponds to a 50.6% and 132.8% increase from the 5th cycle, reflecting increasing irreversibility in electrochemical reactions. The peak shifts in α_n_ and α′_n_ (where “n” is the cycle number) from the 5th cycle (α_5_ and α′_5_) were also monitored to assess lithiation/delithiation stability (Figure 2f). The IEE system showed negligible shifts even after 200 cycles, confirming high reversibility, while QSSE displayed continuous shifts, especially in delithiation peaks, indicating difficulty in lithium extraction from SiMPs due to rising interfacial resistance and contact loss.

Electrochemical impedance spectroscopy (EIS) further revealed the evolution of interfacial resistance, focusing on SEI layer resistance (R_SEI_) and charge transfer resistance (R_ct_). After the initial cycle, the IEE system exhibited significantly lower R_ct_ (24.2 Ω) compared to the QSSE system (51.4 Ω), reflecting a more stable, low‐resistance interface due to robust interlocking (Figure S10, Supporting Information). After 100 cycles, both systems showed increases in R_SEI_ and R_ct_ due to cumulative SEI formation and interface changes. However, the QSSE system exhibited a marked increase in both resistances (R_SEI_: 52.8 Ω, R_ct_: 86.5 Ω), indicating the formation of a thick, nonuniform SEI layer and unstable interface, which increased impedance and degraded Li‐ion kinetics over time. In situ galvanostatic EIS provided real‐time resistance changes during lithiation and delithiation, correlating interface stability with relative capacity. Both systems exhibited a characteristic “w” pattern in EIS profiles, indicative of the Si lithiation process (Figure 2g; Figure S11, Supporting Information).^[^ ^26^ ^]^ In QSSE, R_ct_ was initially high during the early stage of lithiation due to the unstable interface, hindering efficient Li‐ion transport. As lithiation progressed, R_ct_ decreased significantly, which may be attributed to the disruption of the vulnerable SEI layer due to SiMP expansion and the formation of lithiated Si (Li*_x_* Si) which improved Li‐ion kinetics.^[^ ^27^ ^]^ During deep lithiation to Li_15_Si_4_, both R_SEI_ and R_ct_ increased, obstructing further Li‐ion diffusion. In delithiation, the QSSE system initially showed a decrease in resistance until reaching ≈30% relative capacity, after which resistance abruptly increased. This increase signified contact loss, further destabilizing the interface, as the non‐uniform SEI layer continually decomposed and reformed in response to SiMP volume changes, exacerbating Li‐ion transport hindrances and electrical isolation of active materials. In contrast, the IEE system displayed consistently lower R_SEI_ and R_ct_ values, with minimal changes throughout lithiation and delithiation, reflecting the stable and low‐resistance interface provided by the interlocking network, which maintained homogeneous ion/electron transport and suppressed SEI decomposition–reformation cycles seen in the QSSE system.

The morphological evolution of SiMP electrodes was examined via scanning electron microscopy (SEM) in pristine state, after the 1st and 30th cycle (Figure 2h; Figure S12, Supporting Information). The IB/LE system showed drastic volume expansion (81.9%) and severe particle pulverization after the first cycle, attributed to the absence of a flexible buffer layer (Figure S13, Supporting Information). Analogously, the QSSE system exhibited significant swelling after the first cycle, with an increase in thickness of ≈72.6%, while it slightly suppressed the particle pulverization. In contrast, the IEE system showed minimal swelling, with only a 5.3% increase in thickness, indicating effective volume accommodation through the interlocking network. Notably, both the QSSE and IEE systems experienced severe volume expansion during the 1st lithiation, with increases of 295% and 241%, respectively. However, only the IEE system almost fully recovered to its original thickness after delithiation, demonstrating the advantage of its interconnected network. The QSSE system, on the other hand, exhibited microscale voids after the 1st delithiation, confirming its inability to recover and sustain a seamless interface during contraction. This contact loss disrupted uniform ion/electron transport to active Si sites and reduced mechanical support, accelerating mechanical and electrochemical degradation, resulting in capacity fading and low CE. After 30 cycles, the QSSE system showed further structural degradation, with a thickness increase to 18.4 µm (93.7% expansion) and enlarged visible microvoids and cracks, indicating severe aging and interface instability from repeated contact breaking. In contrast, the SiMP electrode in the IEE system displayed a comparatively modest thickness increase of 28.7%, reflecting superior structural retention provided by the interlocking network. The enlarged cross‐sectional SEM image further underscored these differences: the IEE preserved SiMP integrity and maintained a smooth, well‐defined interface, while the QSSE system showed a thick SEI layer, microcracks, and pulverized SiMP particles. These observations highlight the limited ability of the QSSE system to manage the large volume changes inherent to SiMPs, which promoted SEI reformation, particle fragmentation, and interface degradation.

**Figure 3:**
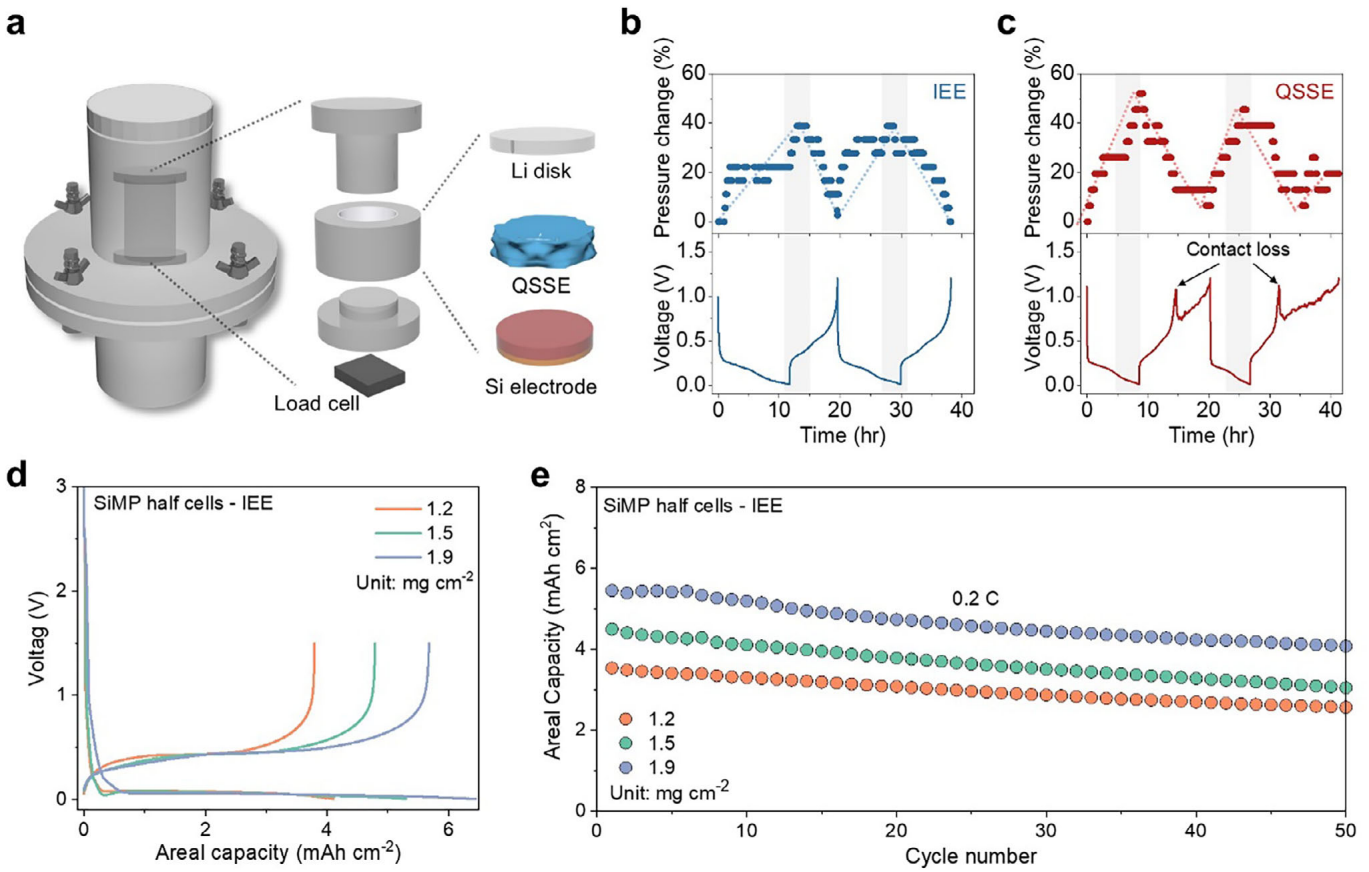
Characterization of Si electrodes half cells with different systems. a) Schematic illustration of a cell integrated with a force sensor and the components in the cell. Pressure changes and voltage profiles for b) IEE system and c) QSSE system. d) Galvanostatic charge–discharge voltage profiles and e) areal capacity retention of SiMP electrode half cells in IEE system with various areal mass loadings.

To gain deeper insights into the interfacial stability of the IEE system, a pressure‐detecting cell kit was utilized to monitor pressure changes through a load cell positioned beneath the kit (**Figure** **3**a). Unlike coin‐type cells, this cell kit captures pressure changes due to volume fluctuations of SiMPs without reverting to its original volume after delithiation, as it lacks a spring component. This setup enables precise tracking of pressure variations directly linked to SiMP volume changes during cycling, providing insights into the interfacial behavior of the system.^[^ ^28^ ^]^ SiMP half cells were cycled under an initial stack pressure of 0.5 Mpa, equivalent to that applied in the 2032 coin‐type cells used in this study.^[^ ^11^ ^]^ During two cycles, the IEE system demonstrated a stable galvanostatic discharge–charge voltage profile with moderate pressure fluctuations, peaking at a 38% increase during lithiation (Figure 3b). This relatively moderate pressure change indicates that the interlocking network effectively buffers SiMP volume expansion, preserving electrode integrity. In contrast, the QSSE system displayed larger pressure changes, reaching up to 52% (Figure 3c). These changes were accompanied by sudden voltage drops and large fluctuations during delithiation, suggesting significant contact loss between the electrode and the QSSE. These voltage fluctuations and pressure changes indicate that the QSSE system struggles to maintain a stable interface as the SiMPs expand and contract, leading to mechanical disconnection and poor electrochemical performance. Additionally, while the IEE system returned back to its initial pressure after delithiation, indicating a reversible response to volume changes, the QSSE system exhibited persistently elevated pressures, confirming its inability to recover to the original configuration. These findings highlight that while the flexibility of QSSEs as a buffering layer is essential, it is insufficient alone; robust interlocking between the electrodes and the QSSE is critical to accommodate volume changes, ensuring stable and repeatable cycling without contact loss.

Building on the excellent electrochemical performance and interface stability observed in the IEE system, its practical viability was further evaluated by testing high areal mass loadings of SiMPs, which are essential for achieving high areal capacity and volumetric energy density in practical battery applications. The IEE system enabled the stable operation of electrodes with an areal mass loading of up to 1.9 mg cm^−2^, corresponding to an areal capacity of ≈5.7 mAh cm^−2^ (Figure 3d). Even at these high mass loadings, SiMP half cells using the IEE system maintained stable cycling over 50 cycles with minimal capacity degradation, without the need for SOC controls (Figure 3e). The robust interlocking network and QSSE flexibility effectively accommodate SiMP volume changes, preventing void formation and contact loss, thereby enabling consistent performance over commercially relevant loadings.

**Figure 4:**
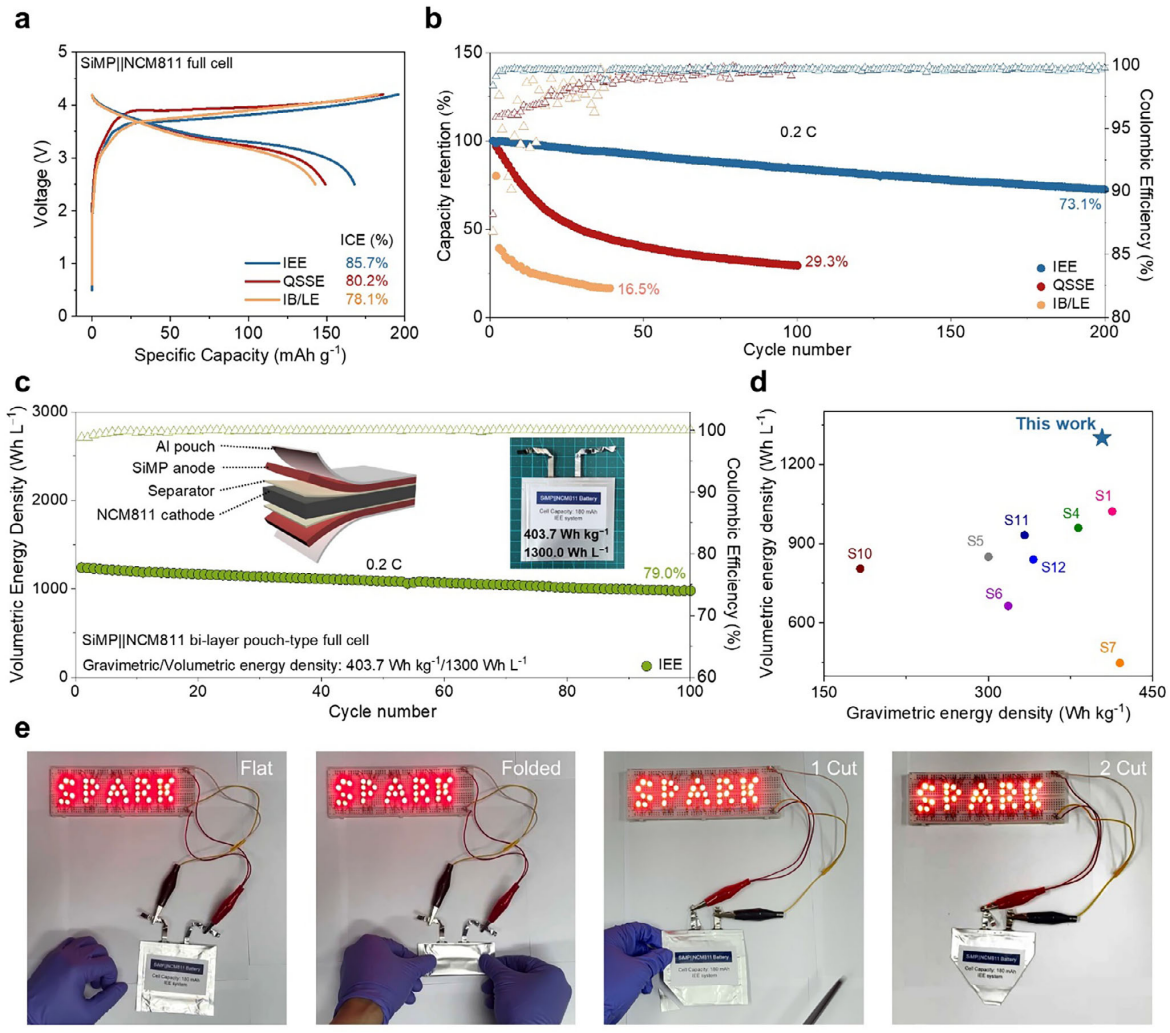
Electrochemical performance of SiMP||NCM811 full cell with IEE system. a) Galvanostatic charge–discharge profiles of the SiMP||NCM811 full cells with different systems. b) Capacity retention and corresponding CEs of the SiMP||NCM811 full cells with different systems at 0.2C (1C = 0.2 A g^−1^). c) Cycle performance of bi‐layer pouch‐type SiMP||NCM811 full cell with IEE system (Inset: Digital photo and cell configuration of a bi‐layer pouch‐type full cell). d) Comparison of the gravimetric and volumetric energy densities of the pouch‐type full cell in this work and previously reported using Si‐based anode full cells. e) Optical images for the LEDs powered by a bi‐layer pouch‐type full cell under various conditions, including flat, folded, cut once, and cut twice.

The performance and practical applicability of the IEE system were validated by assembling SiMP||NCM811 full cells with Ni‐rich layeredcathodes (16 mg cm^−2^) at an n/p ratio (capacity ratio of the anode to cathode) of 1.05. The NCM811 cathodes were also fabricated with the IB to form a cohesive and stable interface with the QSSE, creating a monolithic interlocking system from the SiMP anode through the QSSE to the NCM811 cathode, providing comprehensive interfacial stability. During the formation cycle, the IEE system demonstrated a higher ICE (85.7%) and specific capacity (168.5 mAh g^−1^) compared to the LE and QSSE systems, which exhibited ICEs of 78.1% and 80.2%, respectively, coupled with lower specific capacities of 142.7 and 149.1 mAh g^−1^ (**Figure** **4**a). The specific capacity of the full cells was normalized with the mass of cathode active materials. Long‐term cycling further highlighted the durability of the IEE system, with SiMP||NCM811 full cells retaining 73.1% capacity over 200 cycles without implementing additional measures such as pre‐lithiation steps or SOC control by increasing the n/p ratio (Figure 4b). In stark contrast, the LE and QSSE systems suffered from substantial capacity decay, retaining only 16.5% and 29.3% after 40 and 100 cycles, respectively. The IEE system also achieved rapid CE stabilization (99.5% within three cycles), whereas the QSSE system required 42 cycles to reach 99.0%. The high ICE, rapid CE stabilization, and superior long‐term stability of the full cell using the IEE system underscore the importance of not only having a flexible buffer layer to accommodate volume expansion during lithiation but also ensuring a stable electrode–electrolyte interface that prevents contact loss during the delithiation process. This interlocking mechanism is crucial for achieving high reversibility and consistent cycling performance in SiMP||NCM811 full cells.

The scalability of the IEE system was demonstrated in a bi‐layer SiMP||NCM811 pouch‐type full cell assembled using the IEE system with the same n/p ratio (capacity ratio of the anode to cathode) as coin‐type cells, delivering a reversible capacity of 178.9 mAh and achieving an impressive energy density of 403.7 Wh kg^−1^/1300.0 Wh L^−1^ (Figure 4d; Figure S14, and Tables S2–S4, Supporting Information). The pouch cell exhibited robust cycling stability with an energy density retention of 79.0% after 100 cycles, attributed to limited polarization and stable voltage (Figure 4c; S15, Supporting Information). These results represent a significant step toward realizing commercial high‐energy‐density, stable battery technologies. To validate the practical application and safety of the pouch cell, a series of demonstrations and safety evaluations were performed under real‐world conditions. The pouch cell consistently powered customized light‐emitting diodes (LEDs) in a flat state and maintained operation under mechanical abuses such as folding and partial cutting, with no electrolyte leakage or safety issues (Figure 4e). The ability of QSSE to remain intact under mechanical stress further confirms the practical viability of the IEE system for real‐world applications. Additional tests with a drone as the load assessed the performance of the IEE system under varied mechanical conditions. The drone was charged in both flat and folded states, demonstrating stable functionality regardless of mechanical deformation (Figure S16, Supporting Information). In parallel, flammability tests highlighted the enhanced flame‐retardant properties of the QSSE (Figure S17, Supporting Information). When a separator soaked in highly flammable LE was exposed to an external flame, it ignited immediately and continued burning for over 10 s, resulting in the consequential burning of the supporting Al pouch. On the contrary, QSSE effectively mitigated flammability and leakage risks by immobilizing LE within its crosslinked polymer matrix, self‐extinguished within just 3 s, and preserving the Al pouch. These tests validate the potential of the IEE system for integration into various electronic devices and applications prioritizing flexibility, stability, and safety.

**Figure 5:**
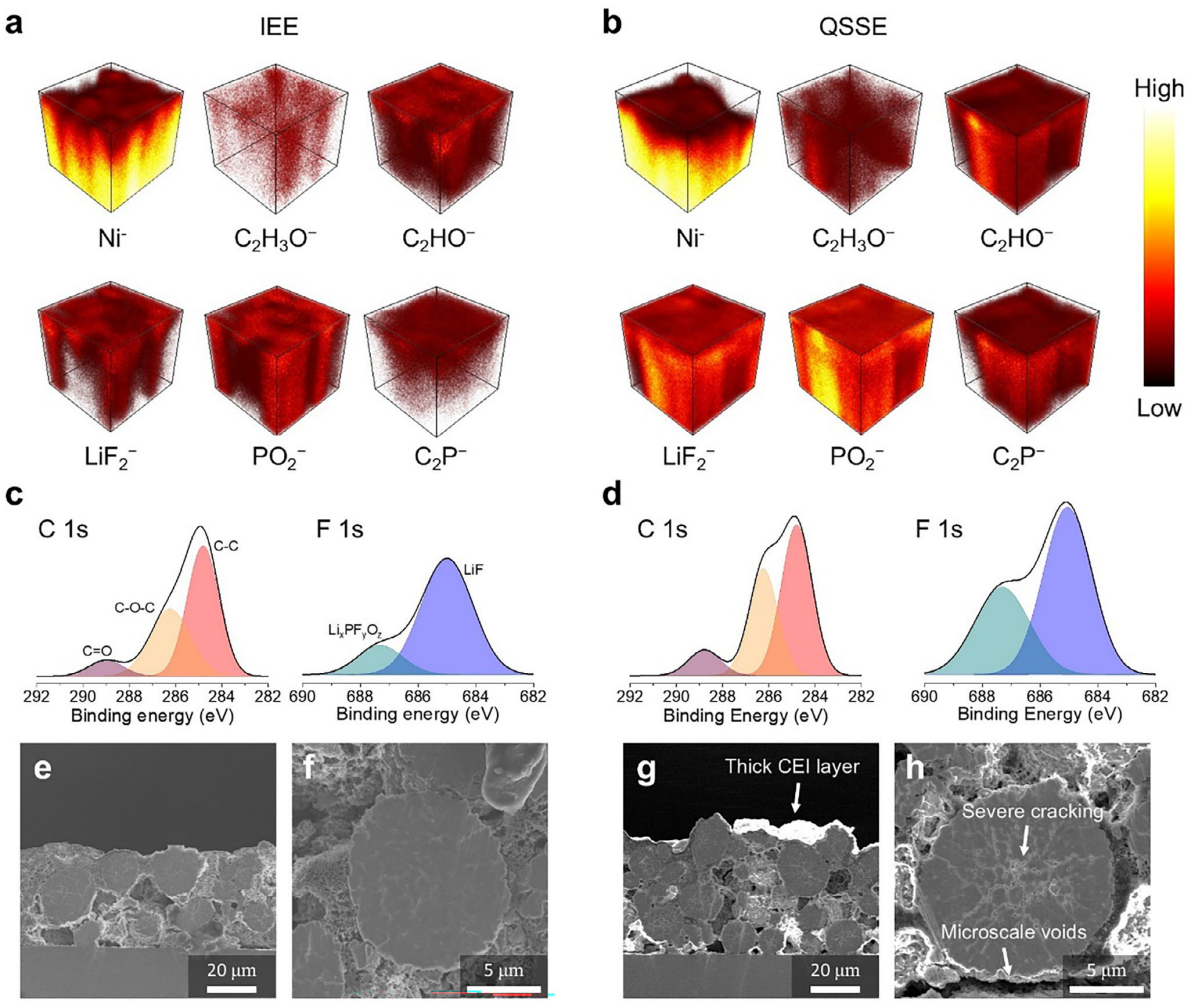
Post‐mortem analysis of NCM811 cathodes with IEE system. TOF‐SIMS 3D reconstruction images of certain species from NCM811 cathodes after 100 cycles of full cells with a) IEE and b) QSSE systems. XPS spectra of C 1s and F 1s of NCM811 cathodes from full cells with c) IEE and d) QSSE systems. Cross‐sectional SEM images of cathodes from full cells with e,f) IEE and g,h) QSSE systems.

A noticeable performance difference was observed between QSSE and IEE systems in full cell configurations, compared to the SiMP half cells. In half cells, the Li metal serves as a relatively soft counter electrode, forming a stable interface with QSSE. However, full cells introduce additional complexities, as both SiMP anodes and NCM811 cathodes face compounded interfacial challenges. Although NCM811 cathodes undergo less volumetric change than SiMP anodes, they still suffer from structural and interfacial instability during cycling. During charging (delithiation), NCM811 particles shrink, leading to the formation of interfacial gaps between the cathode and QSSE, adversely impacting Li‐ion transport and forming a thick and nonuniform CEI layer indicative of degraded kinetics. To validate the effectiveness of the IEE system in maintaining cathode stability, a post‐mortem analysis of NCM811 cathodes was conducted following the full cell test. TOF‐SIMS 3D reconstruction images revealed significant differences in CEI layer formation (**Figure** **5**a,b). In the QSSE system, Ni^−^ fragments were scarcely detected on the NCM811 surface, while high intensities of organic/inorganic CEI components such as C_2_H_3_O^−^, C_2_HO^−^, LiF_2_^−^, PO_2_^−^, and C_2_P^−^ were observed (Figure 5b).^[^ ^29^ ^]^ This accumulation of CEI species suggests the formation of a thick, uneven CEI layer from electrolyte decomposition, which hinders Li‐ion kinetics and contributes to structural degradation. In contrast, the IEE system exhibited a well‐distributed Ni^−^ fragment signal with significantly lower intensities of the aforementioned CEI components, indicating a thin, uniform CEI layer that maintains efficient ion/electron transport (Figure 5a).

X‐ray photoelectron spectroscopy (XPS) further confirmed these findings by comparing the chemical composition of the CEI on the NCM811 cathodes. The C 1s spectra from the IEE system displayed reduced peaks for C−O−C and C = O species, associated with solvent decomposition, compared to the QSSE system, which displayed significantly higher intensities for these peaks (Figure 5c,d).^[^ ^29^, ^30^ ^]^ Similarly, the F 1s spectra of the IEE system showed lower intensities for LiF and Li_x_PF_y_O_z_ peaks, linked to salt and additive decomposition. The TOF‐SIMS and XPS analyses confirm that the IEE system promotes a thin, stable CEI layer on the NCM811 particles, facilitating an interconnected ion/electron transport network, essential for maintaining the electrochemical and structural stability of the cathode during cycling. To investigate the structural integrity of NCM811 particles, cross‐sectional SEM images were obtained using ion‐milling to cut through the electrodes. SEM images revealed that cathodes in the IEE system exhibited a uniform, smooth surface with no visible cracks (Figure 5e,f). In contrast, NCM811 electrodes from the QSSE system displayed nonuniform thickness and a thick, irregular CEI layer on the surface, which caused severe cracking in NCM811 particles (Figure 5g,h). These cracks result from the unstable interface and thick CEI layer, which cause nonuniform lithiation/delithiation.^[^ ^30^ ^]^ To further analyze the CEI layer thickness, high‐resolution transmission electron microscopy (HR‐TEM) was performed on NCM811 cathodes from both the IEE and QSSE systems (Figure S18, Supporting Information). The HR‐TEM images confirm that the IEE system enables the formation of a thin and uniform CEI layer (≈10 nm), which effectively maintains ion transport pathways. In contrast, the QSSE system exhibits a highly nonuniform and thick CEI layer, varying between 10 and 50 nm, which exacerbates interfacial instability. Such findings highlight the challenges of the QSSE system, where unstable and nonuniform CEI formation compromises electrode performance and longevity.

## 3 Conclusion

In summary, an in situ interlocking electrode–electrolyte system is proposed to enhance the stability and performance of QSSBs with SiMP anodes and NCM811 cathodes. Achieved through covalent crosslinking between acrylate groups on a designed IB and a crosslinker within the QSSE, the IEE system creates a robust, interconnected network that effectively mitigates mechanical degradation and maintains stable interfacial contact throughout cycling. Compared to traditional LE and QSSE configurations, which suffer from severe capacity decay due to SiMP pulverization, high interfacial resistance, and unstable SEI formation, the IEE system effectively mitigates these challenges, enabling high capacity retention and stable cycling performance. Notably, the SiMP half cell with the IEE system retained 81.3% of its capacity over 200 cycles, and the SiMP||NCM811 full cell achieved rapid CE stabilization at 99.5% within three cycles. The bi‐layer pouch cell configuration further demonstrated the scalability of the IEE system, delivering a high energy density of 403.7 Wh kg^−1^/1300 Wh L^−1^ and enhanced safety under mechanical abuses, underscoring its potential for high‐energy‐density and durable energy storage applications.

## Conflict of Interest

The authors declare no conflict of interest.

## Supporting information



Supporting Information

## Data Availability

The data that support the findings of this study are available from the corresponding author upon reasonable request.
