# Supplementary material for: Covalently Interlocked Electrode–Electrolyte Interface for High‐Energy‐Density Quasi‐Solid‐State Lithium‐Ion Batteries
Source: Adv Sci (Weinh). 2025 Apr 16;12(22):2417143. doi: 10.1002/advs.202417143 (PMC12165079; doi:10.1002/advs.202417143)
Supplement: Supplementary file 1 — Supporting Information [file ADVS-12-2417143-s001.docx]

Supporting Information

**Covalently Interlocked Electrode-Electrolyte Interface for High-Energy-Density Quasi-Solid-State Lithium-Ion Batteries**

*Dong-Yeob Han, Im Kyung Han, Jin Yong Kwon, Seoha Nam, Saehyun Kim, Youngjin Song, Yeongseok Kim, Youn Soo Kim, Soojin Park*, Jaegeon Ryu**

**Experimental methods**

*Materials preparation*

Polyacrylic acid (PAA) (*M*_n_ = 450,000) was obtained from Polyscience. Metharylic anhydride (MA), Trimethylolpropane propoxylate triacrylate (TMPTA), and pentaerythritol tetraacrylate (PTTA) were purchased from Sigma-Aldirch. Dipentaerythritol hexaacrylate (DPH) was sourced from TCI. Phosphate-buffered saline (PBS, pH = 7.4) was purchased from Thermofisher. 1-Methyl-2-pyrrolidone was obtained from Daejung. Si microparticles (SiMPs) and Li(Ni_0.8_Co_0.1_Mn_0.1_)O_2_ (NCM811) were acquired from Avention and POSCO Future M, respectively.

*Synthesis of Interlocking Binder (IB)*

To synthesize the IB, PAA (2 g) was dissolved in PBS (100 mL) by heating at 60 ℃. MA (4 mL) was added dropwise with stirring, and the reaction was allowed to proceed for 6 hr at 50 ℃. The reaction mixture was then dialyzed against distilled water for 3 days. The resulting product was lyophilized to obtain a white foam.

*Preparation of Quasi-Solid Gel (QSG)*

IB, TMPTA, and BPO were dissolved in a mixed solvent of distilled water and an organic solvent in a 2:8 weight ratio, with the component weight ratios of IB:TMPTA:BPO at 1:6:0.2:93. The precursor solution was heated at 60 ℃ for 1 hr to form the QSG. Control samples included QSG with only crosslinker (TMPTA:BPO:Solvent = 6:0.2:94) and QSG with PAA and crosslinker (PAA:TMPTA:BPO:Solvent = 1:6:0.2:93).

*Materials Characterization*

^1^H NMR spectra were recorded on a Bruker Avance 400 spectrometer at 25 °C and 500 MHz with a delay time was set to 2.5 s. All polymer samples were prepared in D_2_O. In situ NMR measurements were taken every 2 min for 1 hr at 60 °C. Rheological strain amplitude sweeps were performed from 1% to 500% strain at 1 rad s^−1^, while frequency sweeps spanned from 0.1 to 100 rad s^−1^ at a 1% strain. Glass transition temperatures (*T_g_*) were measured using DSC (DSC 4000, PerkinElmer) under an Ar atmosphere. FT-IR (Cary 600, Agilent Technologies) was used for chemical structure analysis. SiMP size distribution was assessed using a laser scattering particle size analyzer (LA-960). TOF-SIMS 3D profiling and depth analysis were conducted with TOF-SIMS 5 (ION TOF). Morphological structures were observed by SEM (S-4800, Hitachi), with ion-milling (Hitacho IM4000) employed for precise electrode cross-sections. Surface chemistry was examined by ex situ XPS (K-Alpha, Thermo Scientific)

*Electrochemical Measurements*

A slurry coating method was employed to prepare the SiMP anodes and NCM811 cathodes. For the anode, SiMPs, binder, and Super P (carbon black) were mixed in a 60:20:20 mass ratio. The cathode was composed of NCM811, binder, and Super P in a 90:5:5 ratio. IB was used as the binder for the IEE system, while PAA served as the binder for the QSSE system. The slurries were cast onto Cu and Al foils for the anode and cathode, respectively. The mass loading of SiMP electrodes ranged from 0.7-1.9 mg cm^-2^, with 1.2 mg cm^-2^ used for full cell test. The NCM811 electrode loading was 15.0-16.0 mg cm^-2^. After casting, the electrodes were dried at 70 ℃ for 12 hr under vacuum. The prepared electrodes were cut into disks and assembled into the CR2032 cells (Welcos) in an Argon-filled glove box using a Celgard 2400 separator, Li metal counter electrode, and electrolytes. LE consisted of 1m LiPF_6_ in EC/EMC/DMC (1:1:1 by volume) + 5wt% FEC (Soulbrain). QSSE was prepared by adding 6 wt% crosslinker (TMPTA) and 0.2 wt% initiator to LE. Crosslinking for QSSE and IEE was completed by thermal curing at 60 ℃ for 1 hr. Galvanostatic battery tests were conducted with cut-off voltage of 0.005-1.5 V for the formation cycle at 0.05 C and 0.01-1.2 V for subsequent cycles at 0.1-3 C on a battery cycler between 2.5 and 4.2 V, with a 0.05 C formation cycle and subsequent cycles at 0.2 C. The specific capacity of the full cells was normalized with the mass of cathode active materials. Ex situ electrochemical impedance spectroscopy (EIS) measurements were conducted between 100 kHz and 0.1 Hz using potentiostatic EIS with a 10 mV amplitude (VSP-300, BioLogic). Ionic conductivity was measured using EIS over a frequency range from 1 MHz to 100 mHz. Lithium transference numbers were determined by performing EIS measurements before and after chronoamperiometry at 0.01 V over the same frequency range (1 MHz to 100 mHz). Oxidation stability was evaluated via linear sweep voltammetry at a scan rate of 1 mV s^-1^ from 3 to 6 V.

*In situ EIS measurement*

Galvanostatic EIS was performed at a constant current of 0.5 C during lithiation and delithiation, with impedance spectra recorded every 12 min. The potentiostat system for in situ EIS used two channels: one for impedance spectra acquisition and another for voltage profile recording. Sinusoidal current waves with a 10 mA amplitude were applied across frequencies from 100 kHz to 0.01 Hz (VSP-300, BioLogic).

*Pressure-detecting cell fabrication*

The pressure-detecting cell was fabricated using a KP-solid cell kit (Welcos) in an argon-filled golve box. A load cell was positioned beneath the lower body of the cell, with a sensor connected to an indicator. Inside the cell kit, a Celgard 2400 separator disk with electrolyte was placed between the SiMP electrode and Li metal disks. The cell was pressed to approximately 0.3 MPa and underwent thermal crosslinking at 60 ℃ for 1 hr. Cycling was conducted to observe changes in the half-cell configuration.

*Bi-layer pouch-type cell fabrication*

The bi-layer pouch-type full cell was fabricated in a dry room with a dew point below -60 ℃. SiMP and NCM811 electrodes were prepared in dimensions of 5.2 × 6.2 cm^2^ and 5.0 × 6.0 cm^2^, respectively. The gravimetric and volumetric energy density of the pouch-type full cell was calculated to be 403.7 Wh kg^-1^ and 1300.0 Wh L^-1^. Detailed calculations for energy densities are provided in Tables S2 and S3.


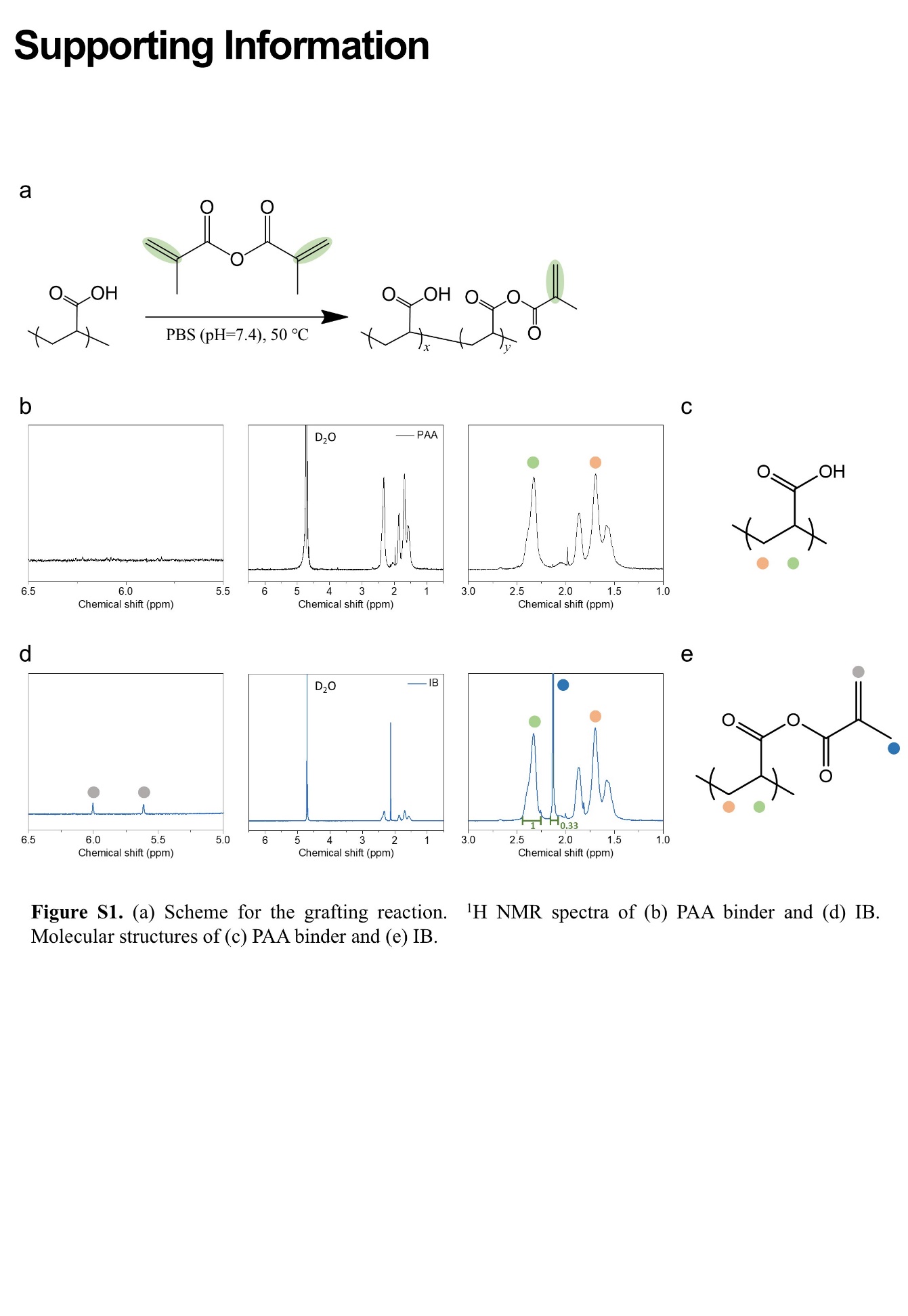


**Figure S1.** (a) Scheme for the grafting reaction. ^1^H NMR spectra of (b) PAA binder and (d) IB. Molecular structures of (c) PAA binder and (e) IB.


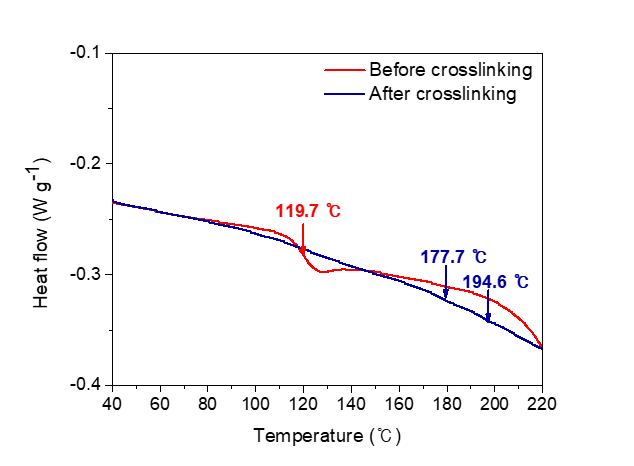


**Figure S2.** DSC curves of IB + Crosslinker before and after crosslinking.


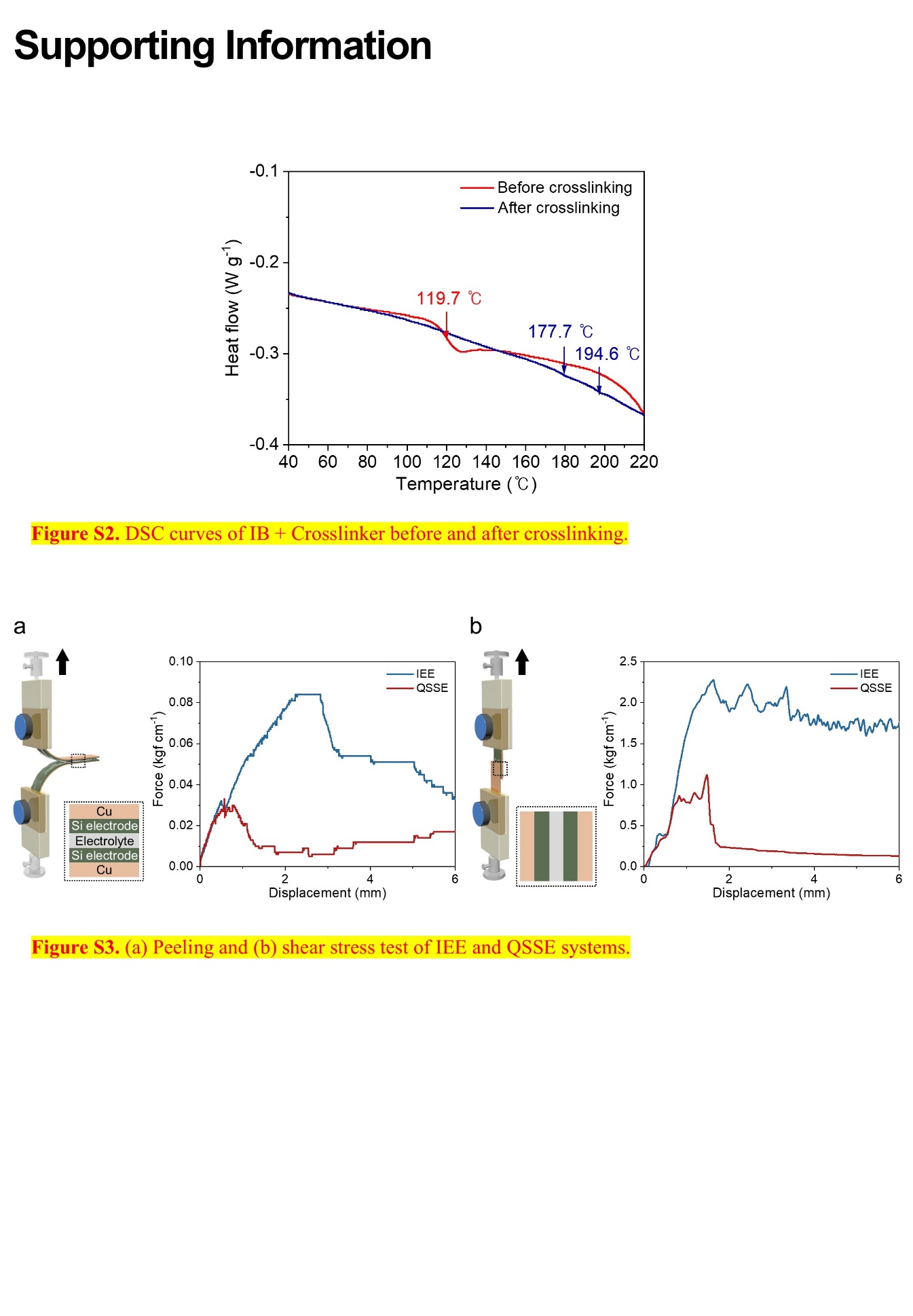


**Figure S3.** (a) 180^o^ peel-off and (b) shear stress test of IEE and QSSE systems.


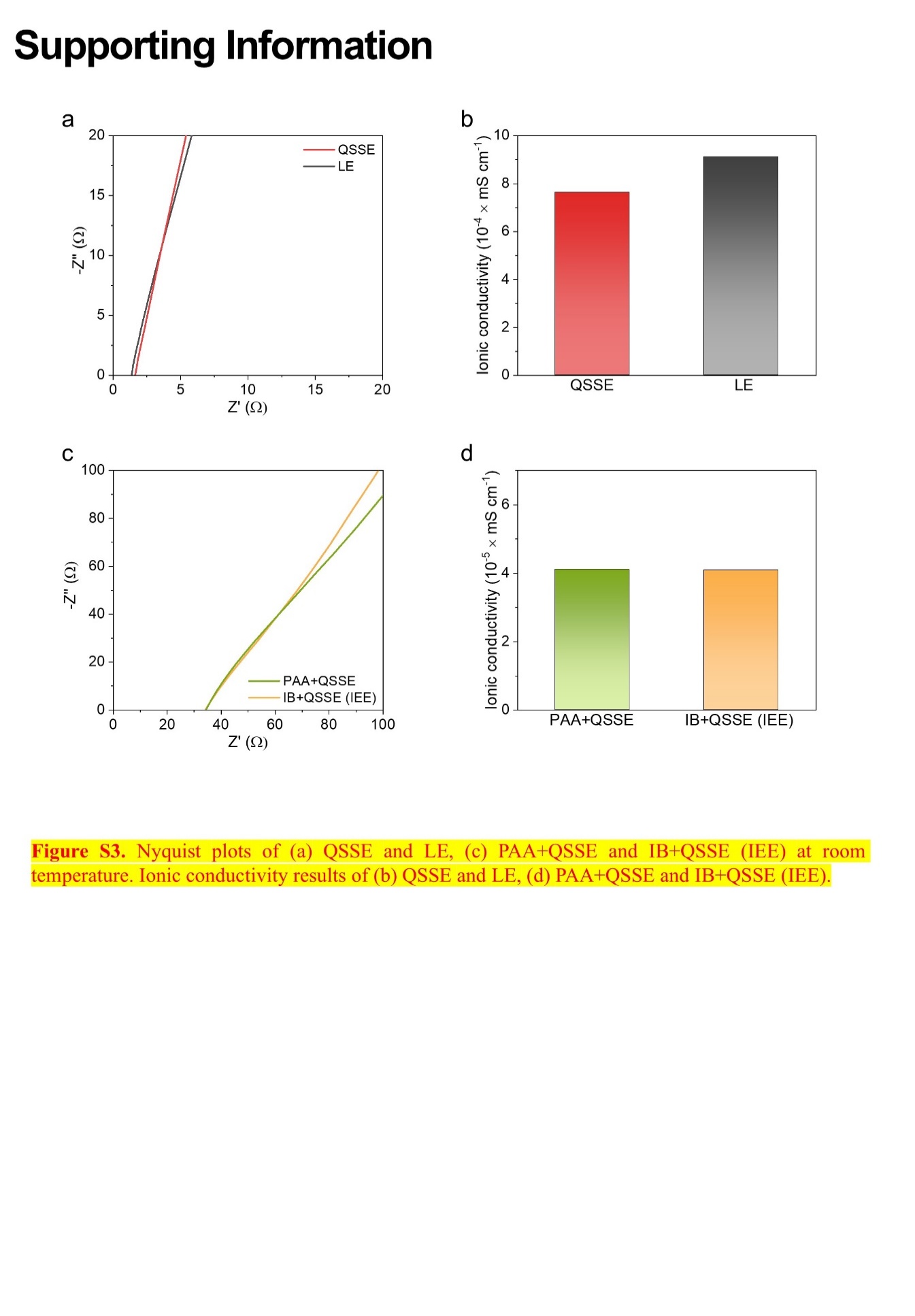


**Figure S4.** Nyquist plots of (a) QSSE and LE, (c) PAA+QSSE and IB+QSSE (IEE) at room temperature. Ionic conductivity results of (b) QSSE and LE, (d) PAA+QSSE and IB+QSSE (IEE).


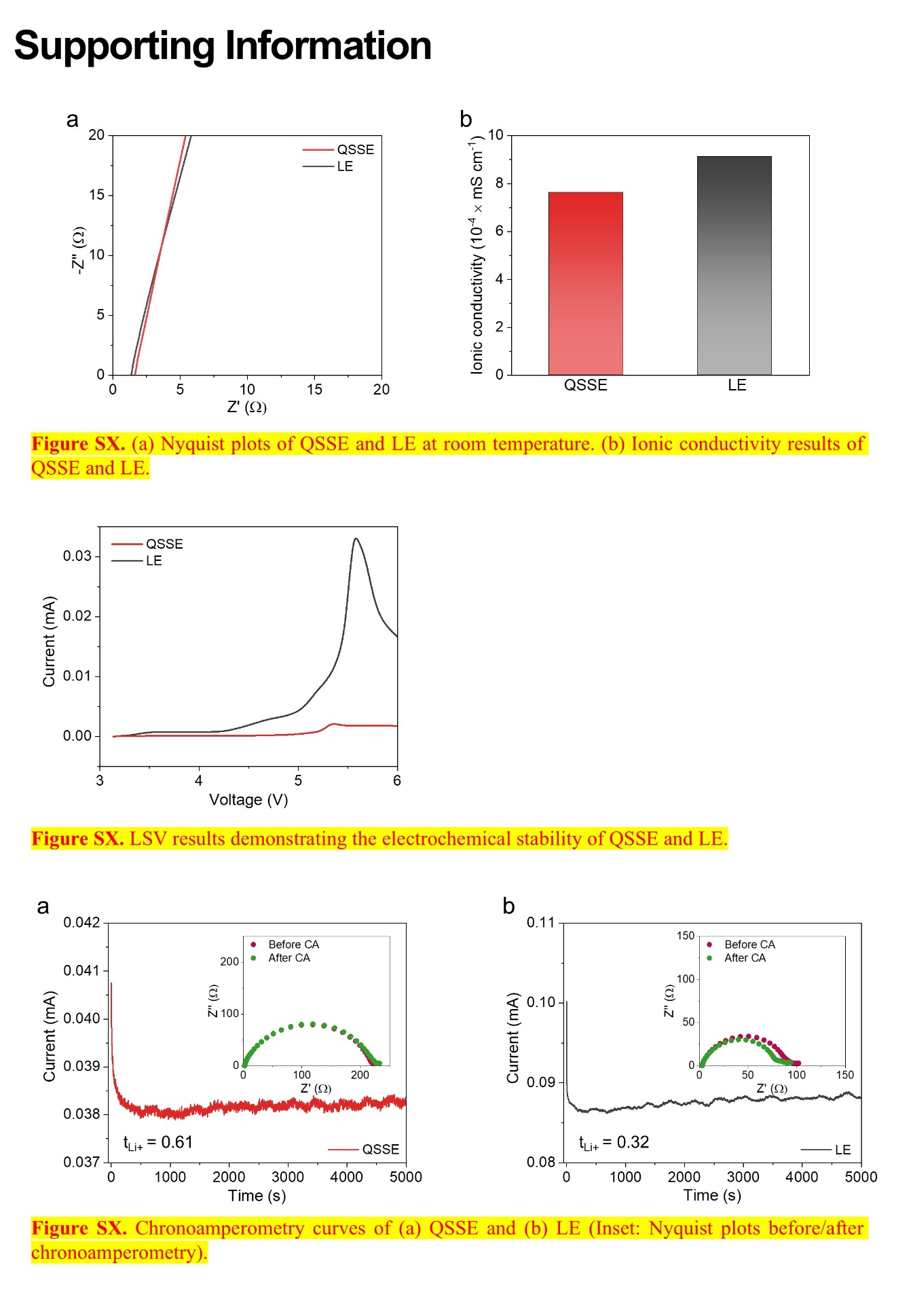


**Figure S5.** Chronoamperometry curves of (a) QSSE and (b) LE (Inset: Nyquist plots before/after chronoamperometry).


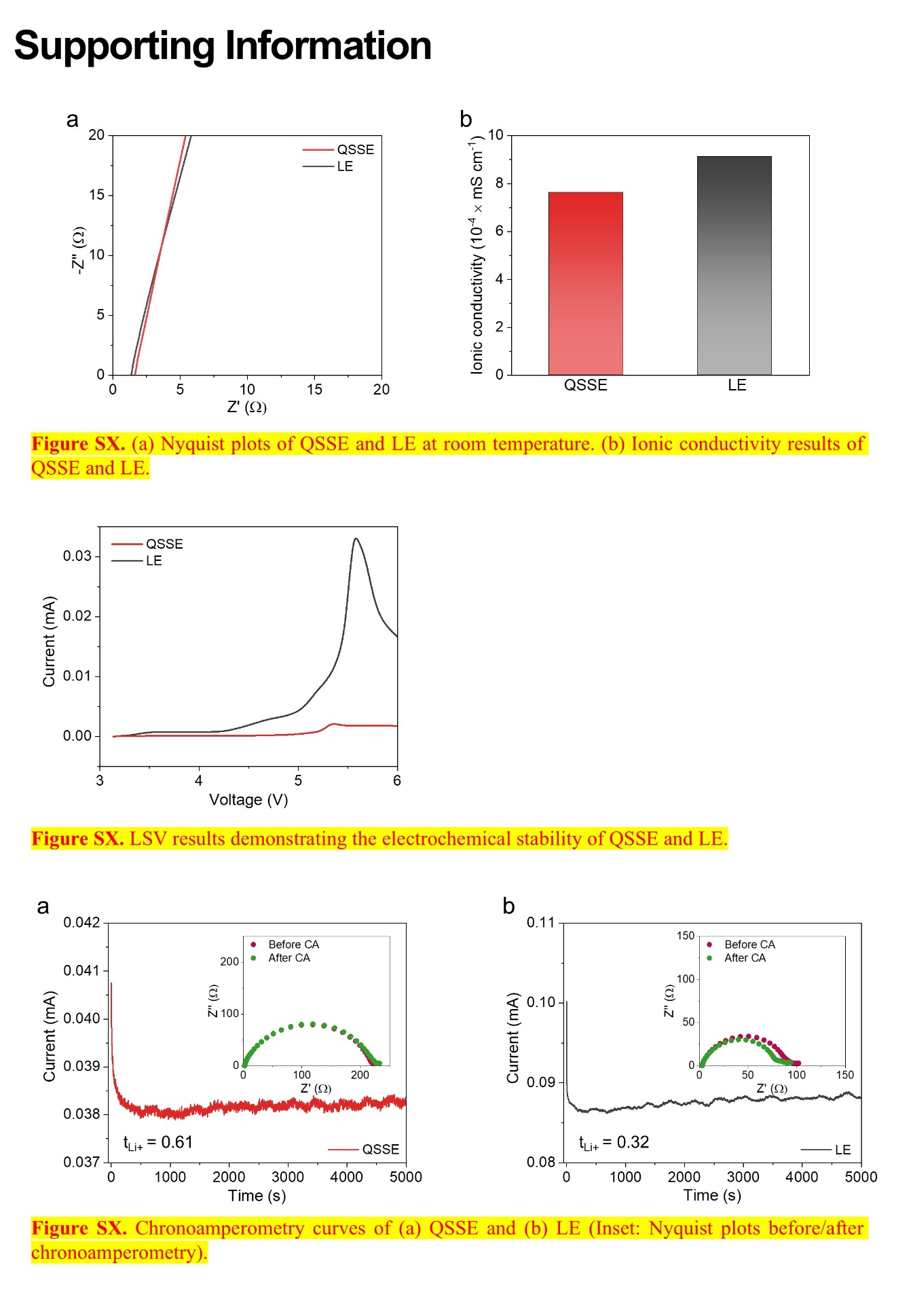


**Figure S6.** LSV results demonstrate the electrochemical stability of QSSE and LE.


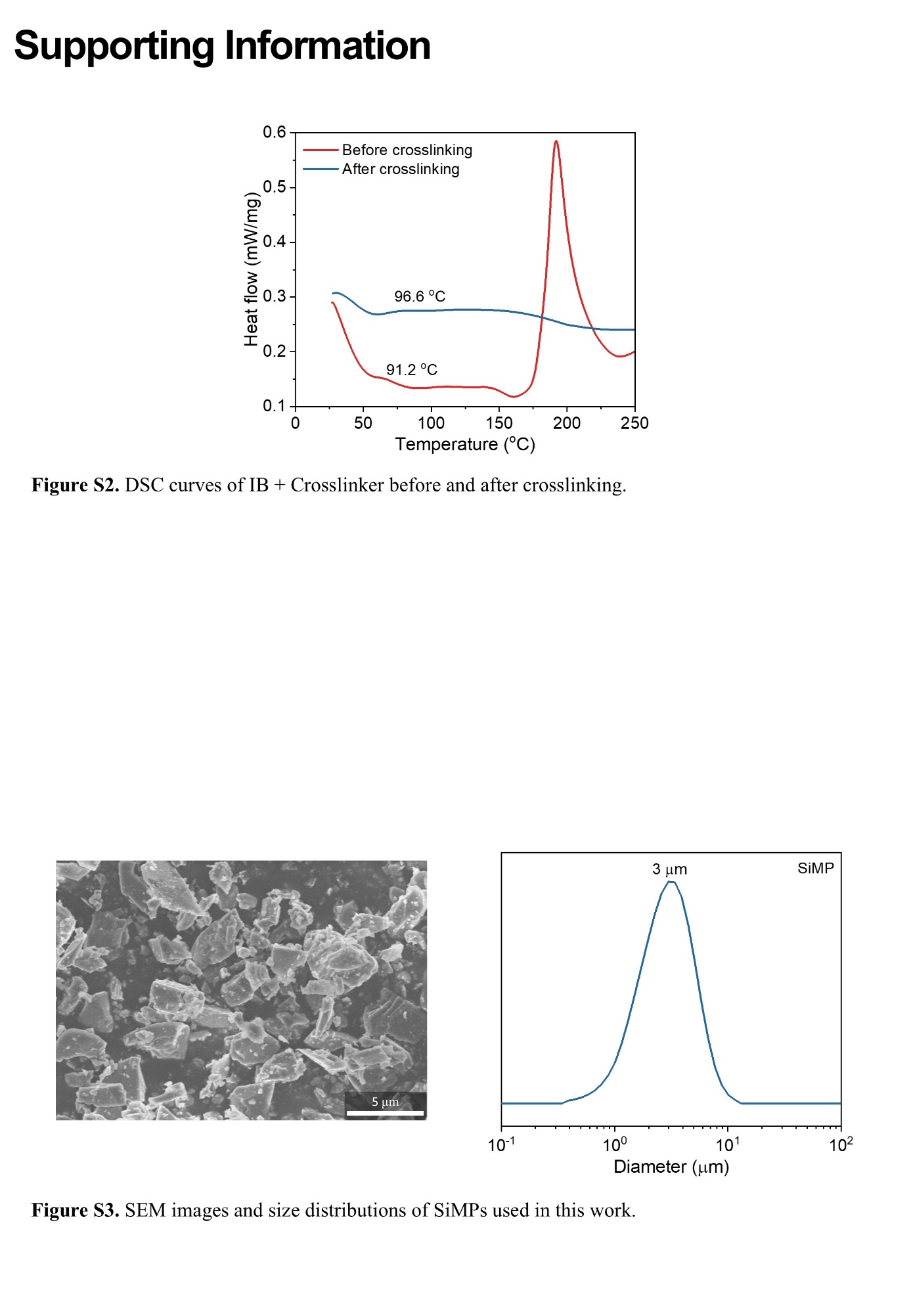


**Figure S7.** SEM images and size distributions of SiMPs used in this work.


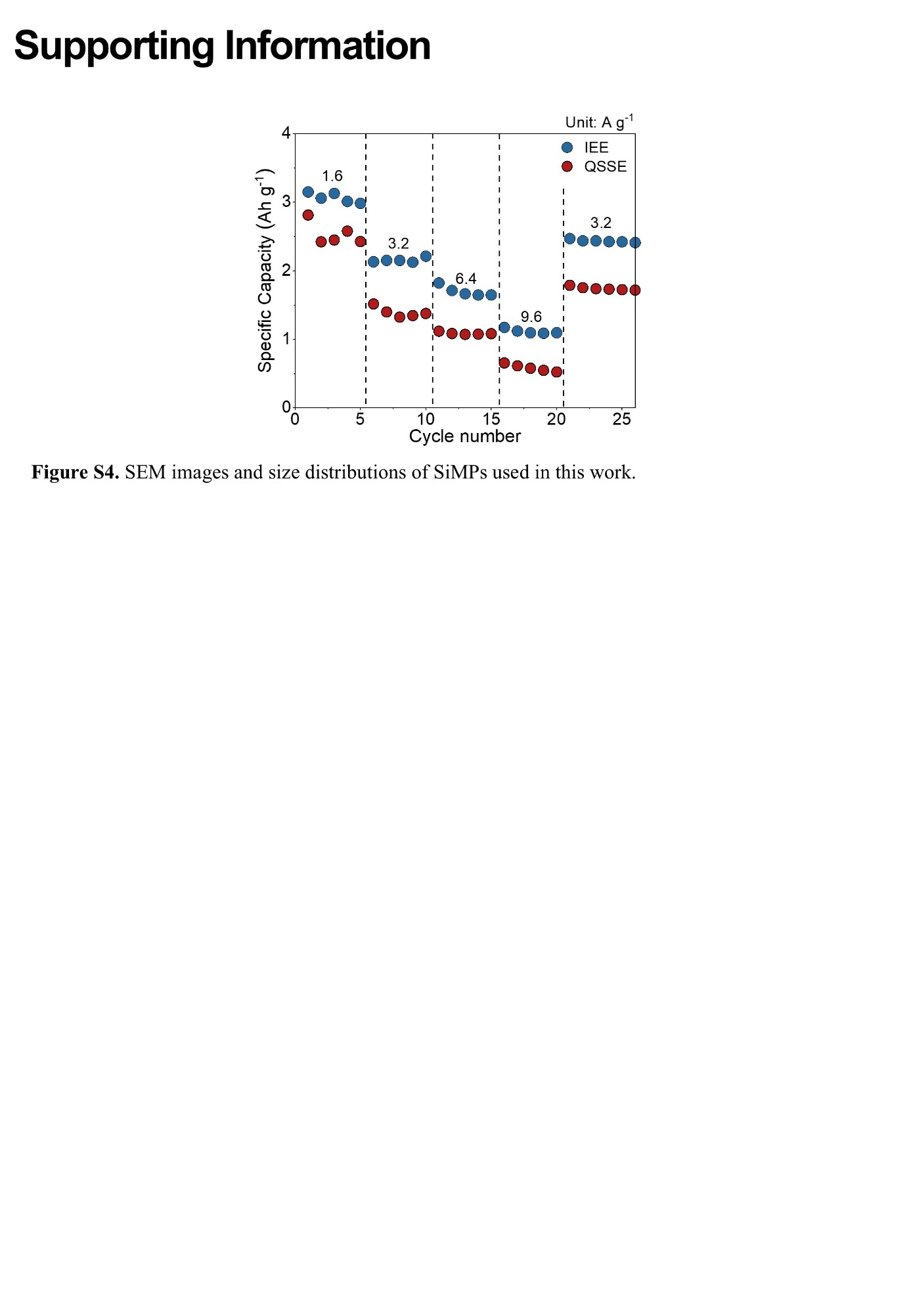


**Figure S8.** Rate capabilities at various current densities of Si electrodes with different systems.


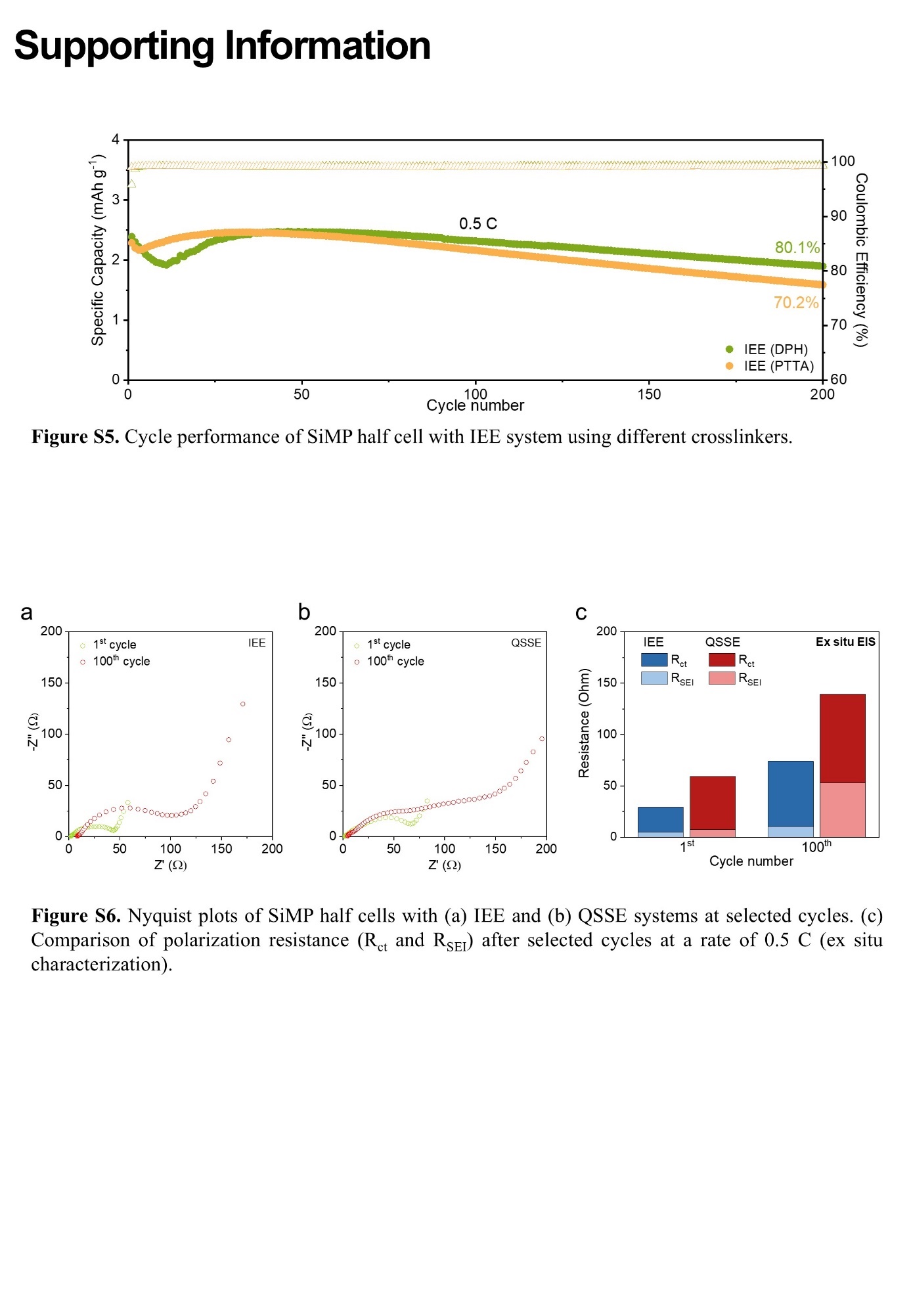


**Figure S9.** Cycle performance of SiMP half cell with IEE system using different crosslinkers.


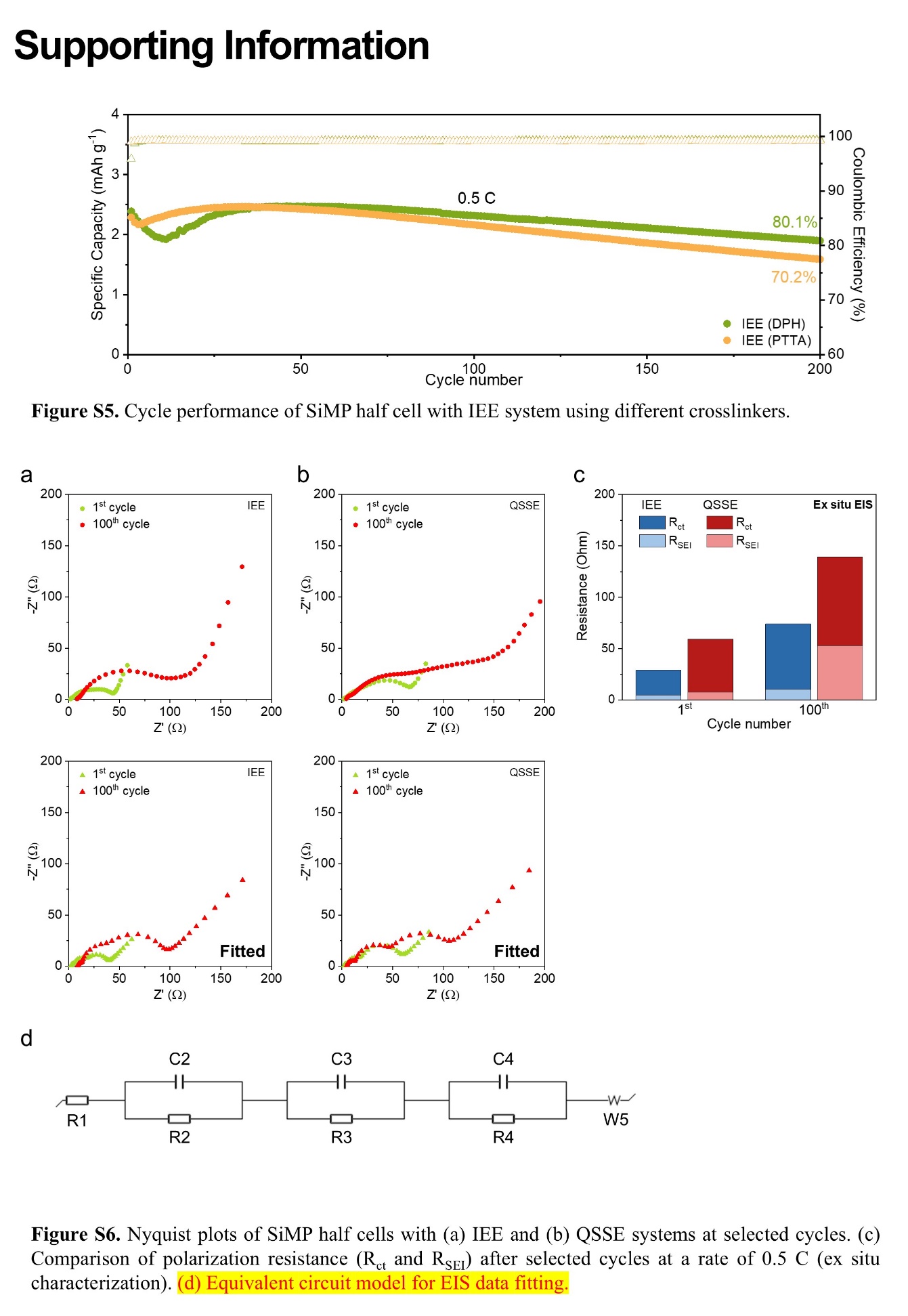


**Figure S10.** Nyquist plots of SiMP half cells with (a) IEE and (b) QSSE systems at selected cycles. (c) Comparison of polarization resistance (R_ct_ and R_SEI_) after selected cycles at a rate of 0.5 C (ex situ characterization). (d) Equivalent circuit model for EIS data fitting.


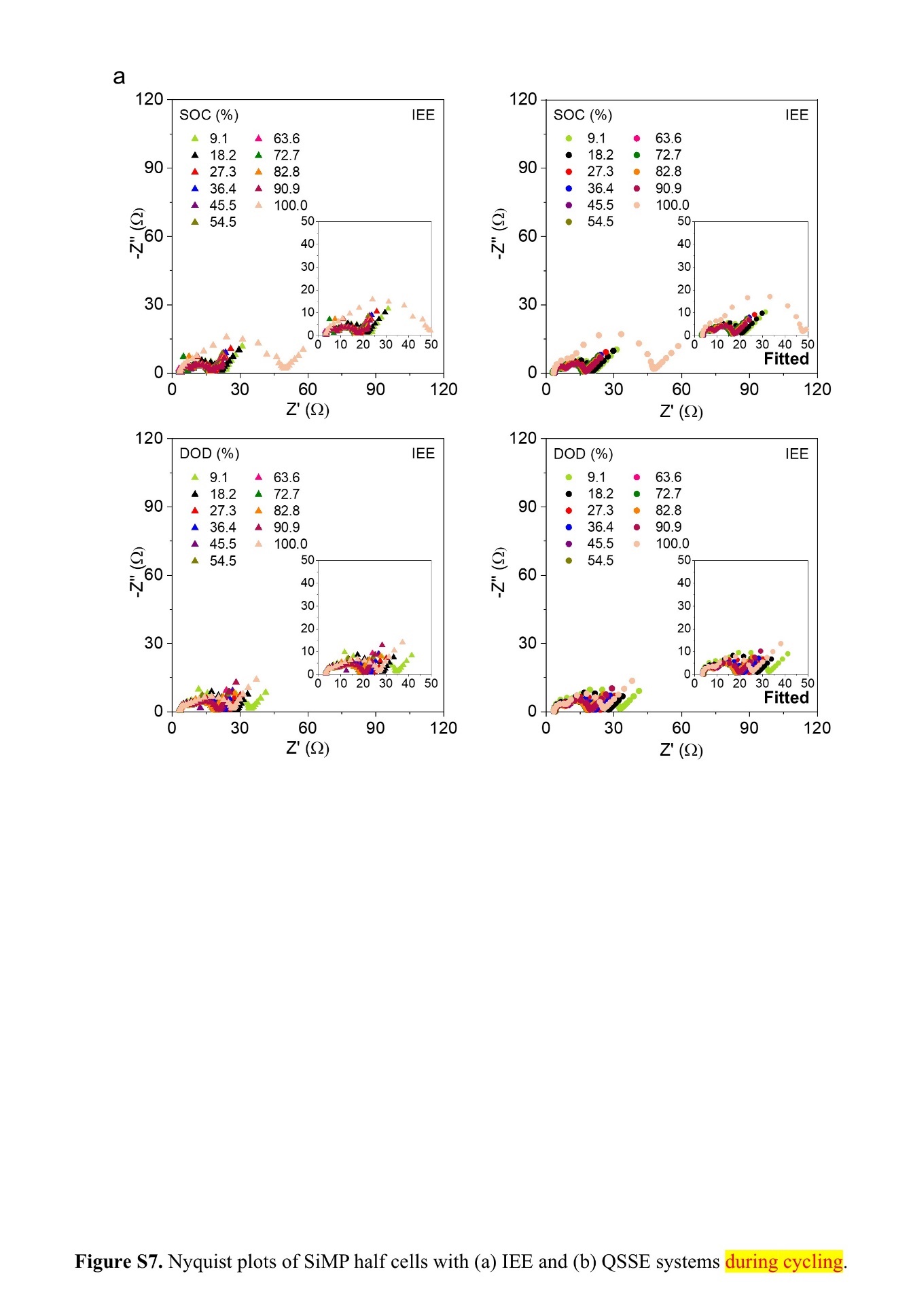


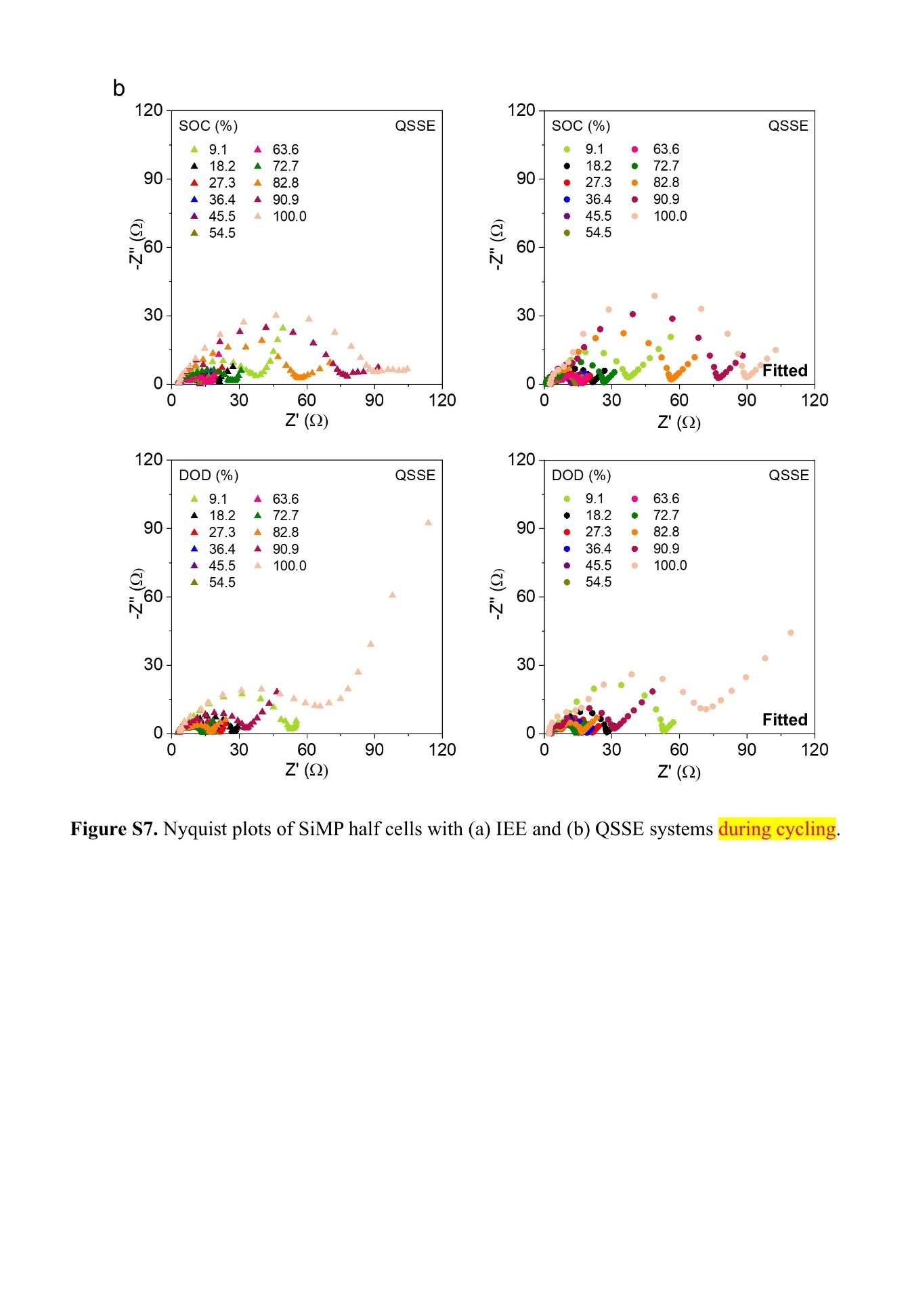


**Figure S11.** Nyquist plots of SiMP half cells with (a) IEE and (b) QSSE systems during cycling.


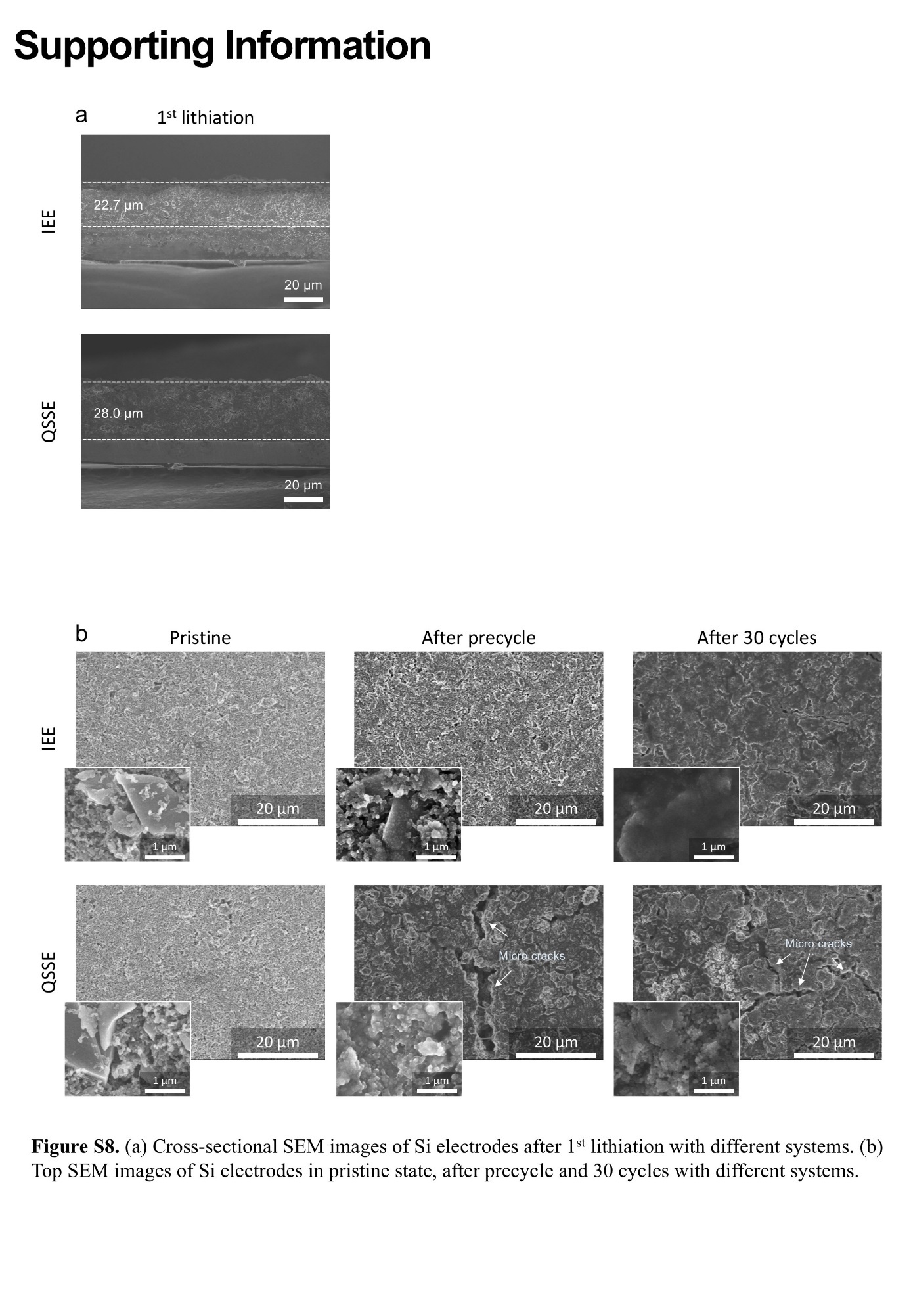


**Figure S12.** (a) Cross-sectional SEM images of Si electrodes after 1^st^ lithiation with different systems. (b) Top SEM images of Si electrodes in pristine state, after precycle and 30 cycles with different systems.


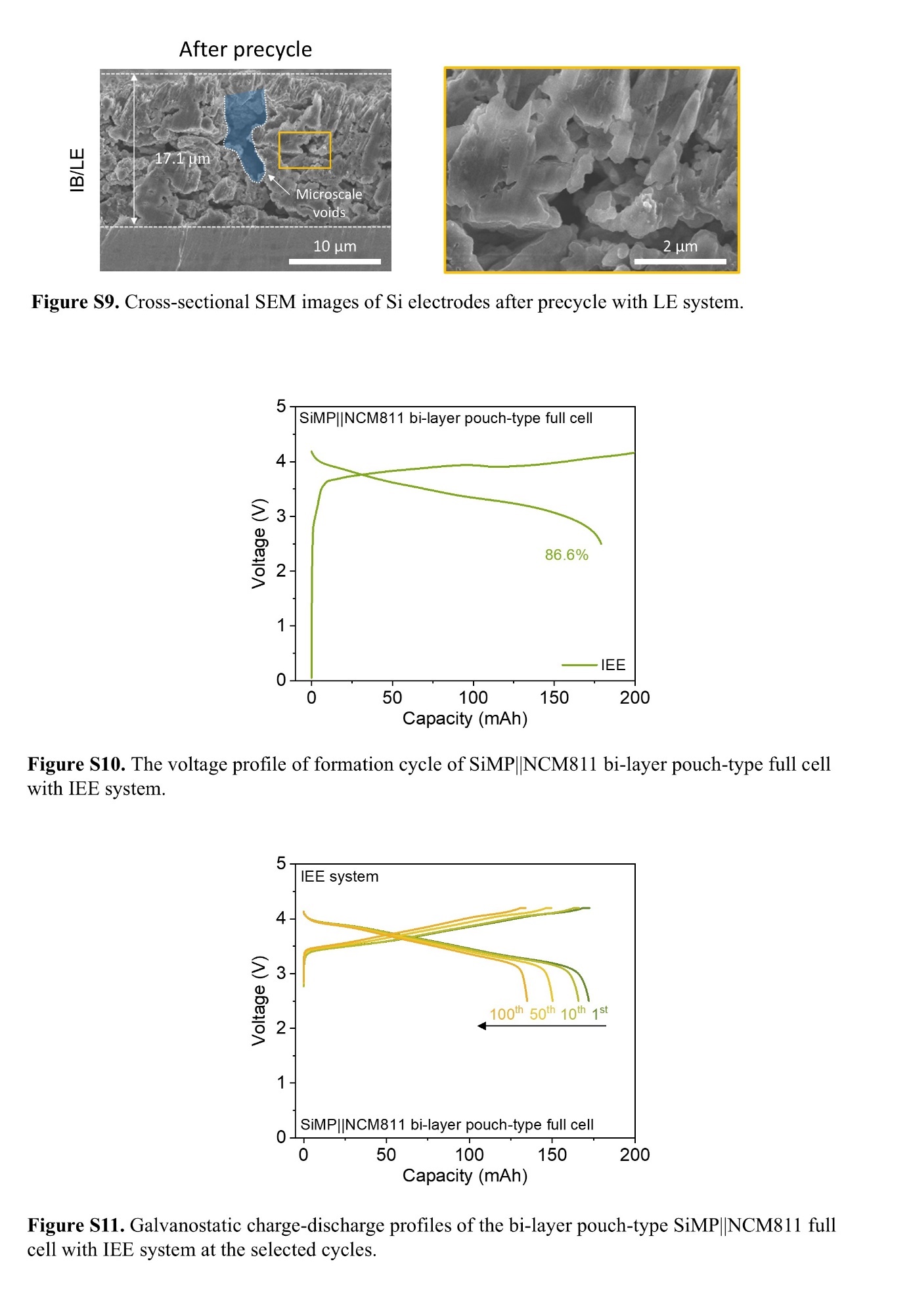


**Figure S13.** Cross-sectional SEM images of Si electrodes after precycle with LE system.


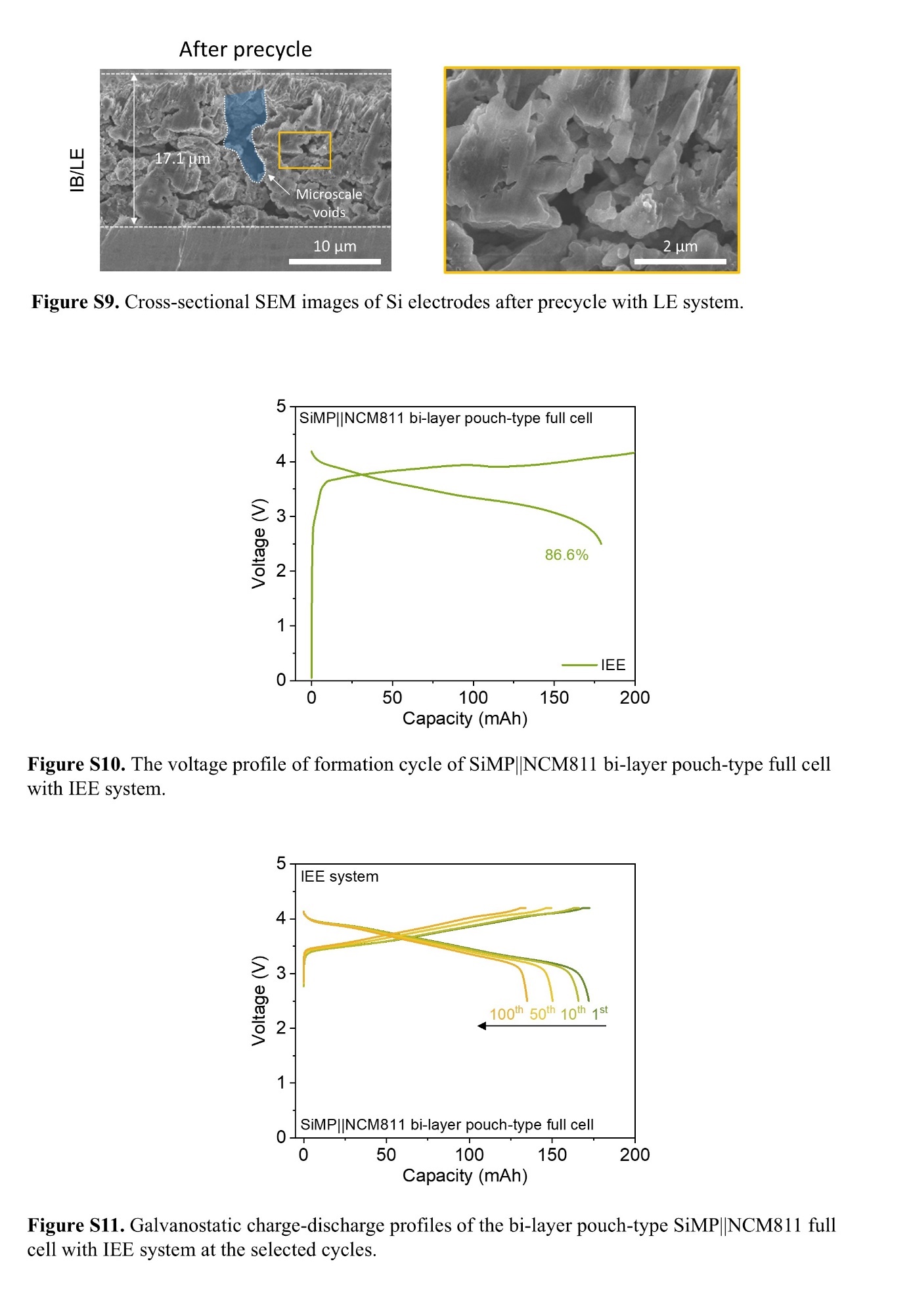


**Figure S14.** The voltage profile of formation cycle of SiMP||NCM811 bi-layer pouch-type full cell with IEE system.


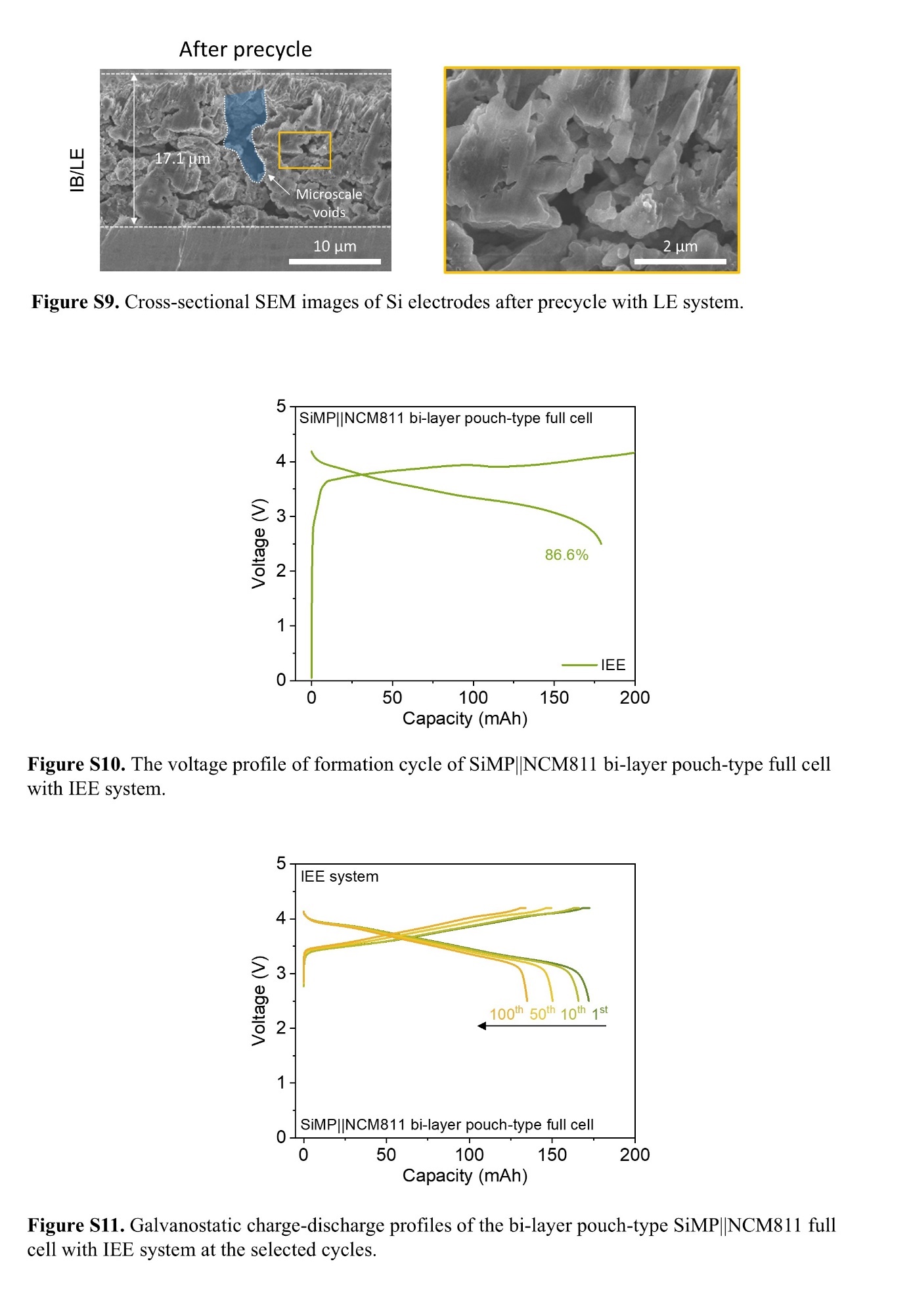


**Figure S15.** Galvanostatic charge-discharge profiles of the bi-layer pouch-type SiMP||NCM811 full cell with IEE system at the selected cycles.


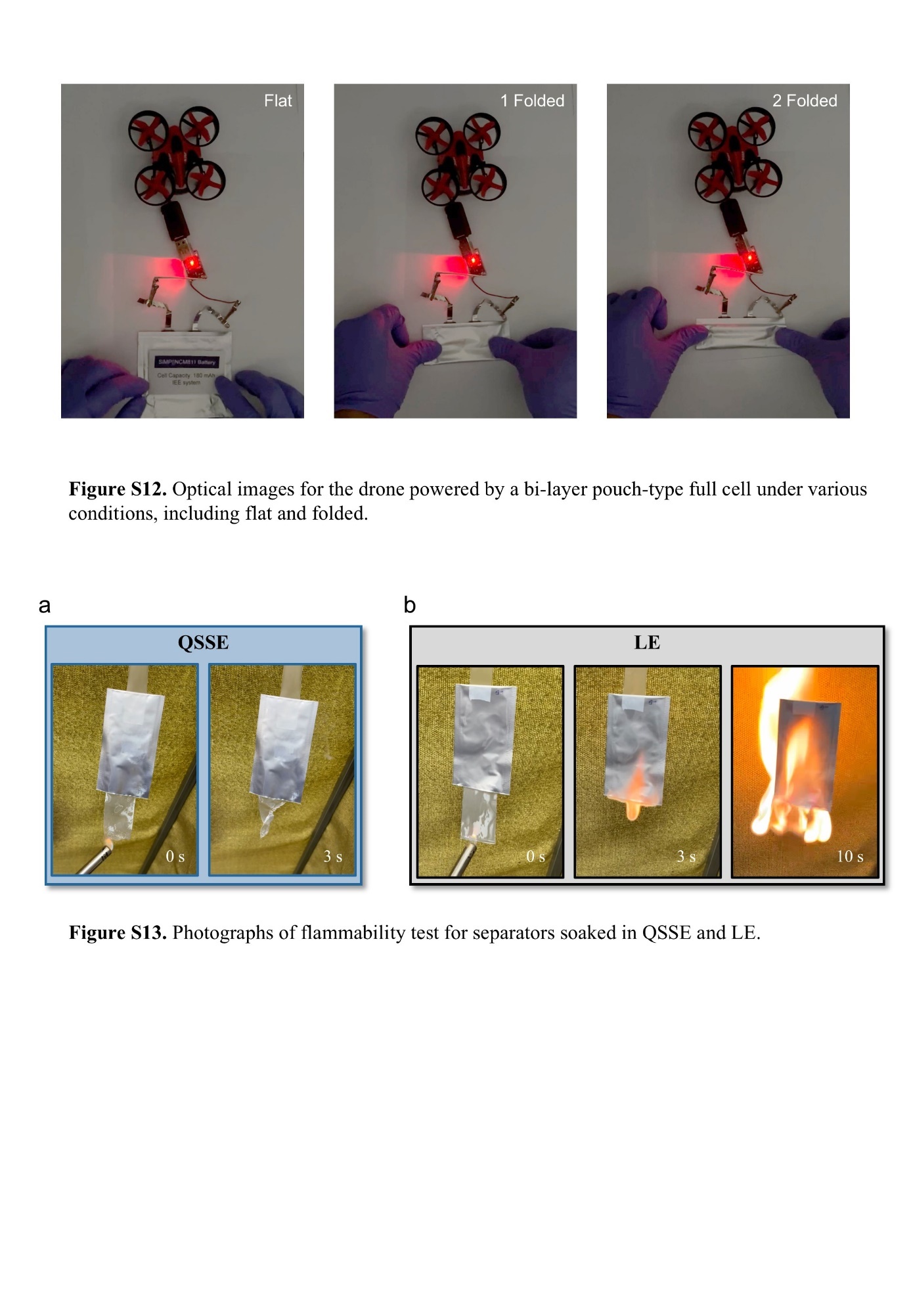


**Figure S16.** Optical images for the drone powered by a bi-layer pouch-type full cell under various conditions, including flat and folded.


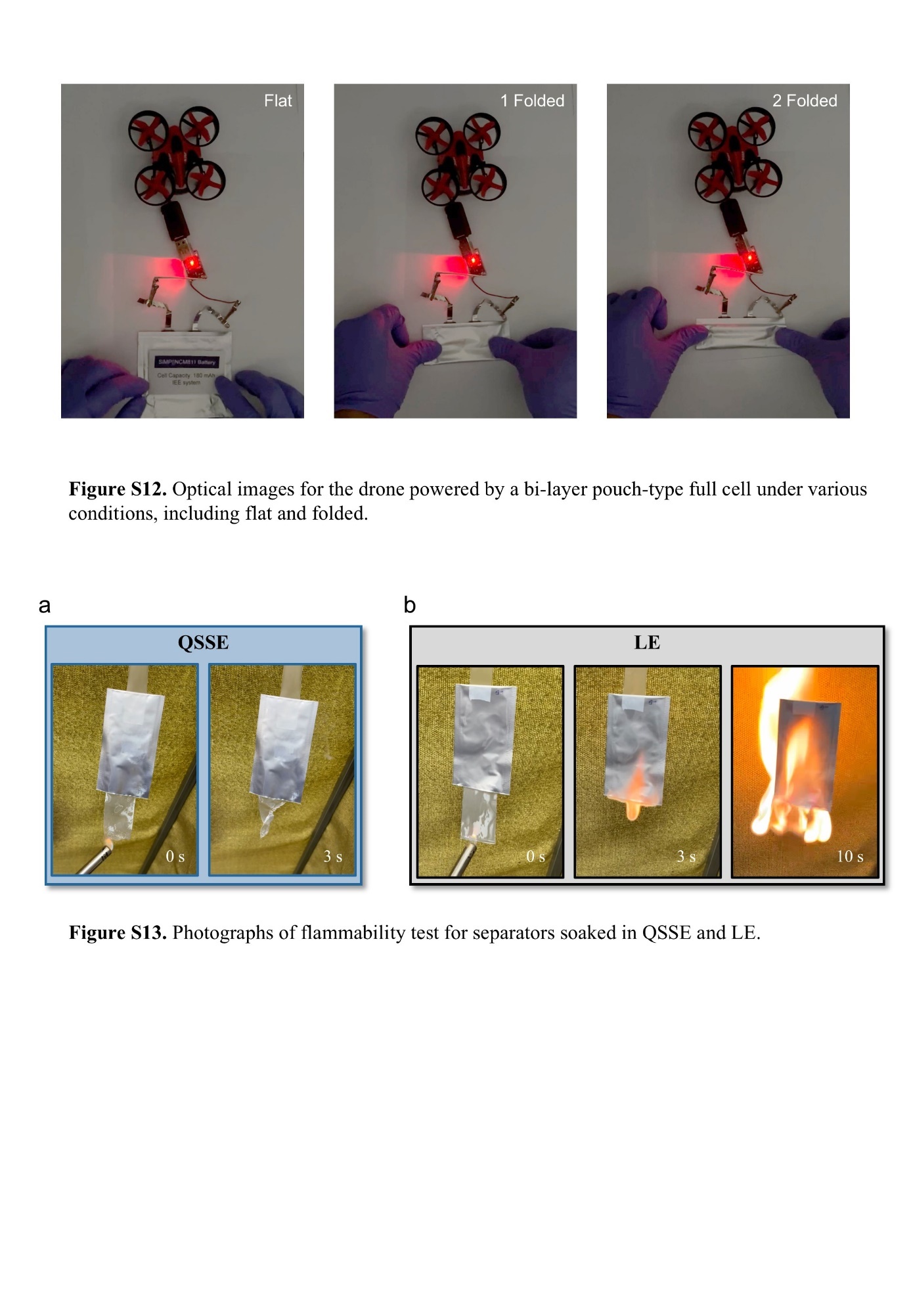


**Figure S17.** Photographs of flammability test for separators soaked in QSSE and LE.


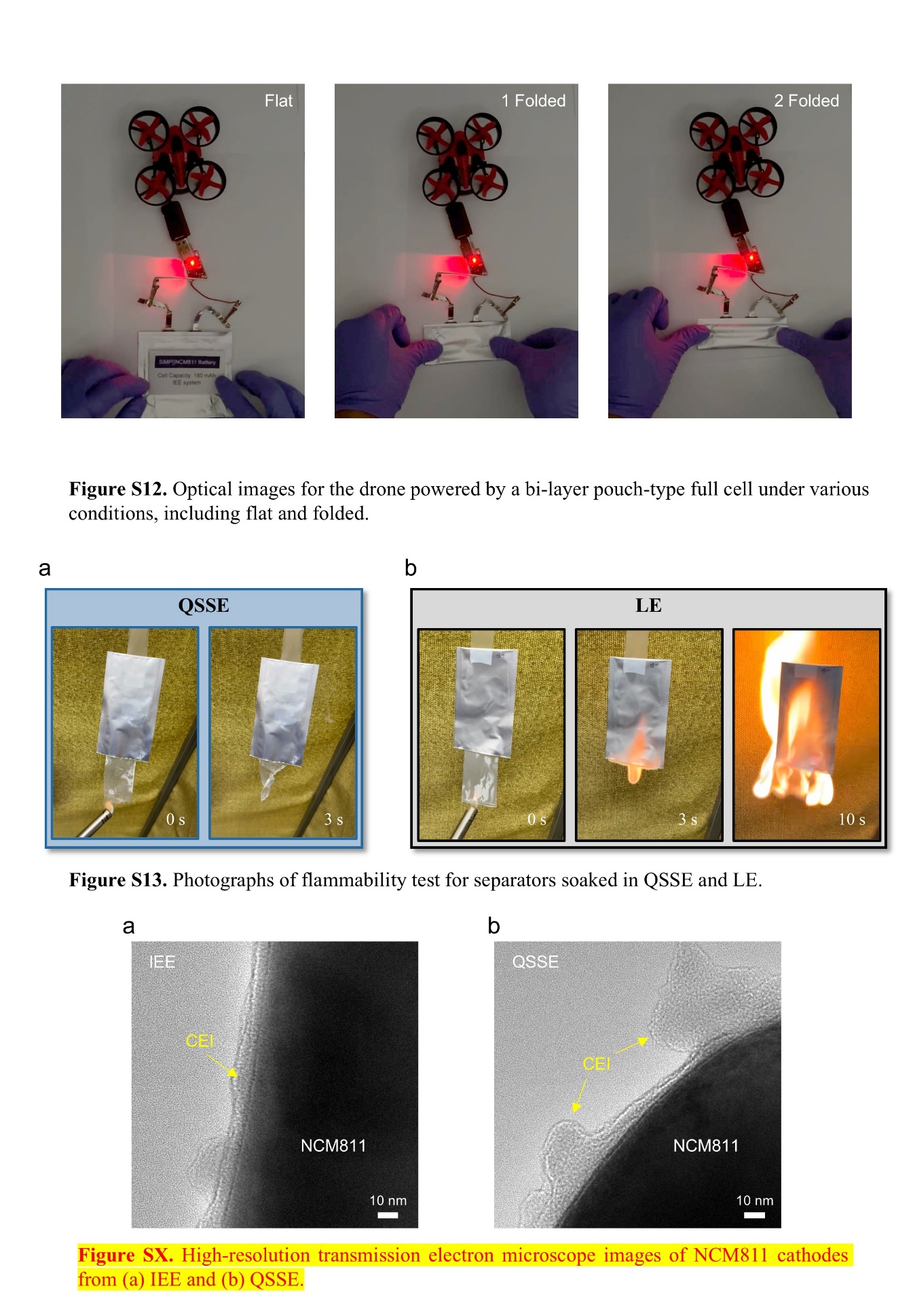


**Figure S18.** High-resolution transmission electron microscope images of NCM811 cathodes from (a) IEE and (b) QSSE.

**Table S1.** Table of discharge, charge capacities, and ICE of formation cycle of SiMP half cells with different systems.

|  | Discharge capacity  (Ah g^-1^) | Charge capacity  (Ah g^-1^) | ICE  (%) |
| --- | --- | --- | --- |
| IEE | 3.895 | 3.606 | 92.6 |
| QSSE | 3.601 | 3.264 | 90.7 |
| IB/LE | 3.455 | 2.944 | 85.2 |
| PAA/LE | 3.738 | 3.357 | 89.8 |

**Table S2.** Calculation details for the gravimetric energy density of the bi-layer pouch-type full cell using IEE system based on electrodes (top) and essential components (bottom).

| Cu foil  (mg) | Anode  (mg) | Al foil  (mg) | Cathode  (mg) | Total electrode mass  (mg) | Capacity  (mAh) | Working voltage  (V) | Gravimetric energy density  (Wh kg^-1^) |
| --- | --- | --- | --- | --- | --- | --- | --- |
| 562 | 68 | 87.9 | 921.6 | 1639 | 178.9 | 3.7 | 403.7 |

| Total electrode mass  (mg) | Separator  (mg) | Electrolyte  (mg) | Total mass  (mg) | Capacity  (mAh) | Working voltage  (V) | Gravimetric energy density  (Wh kg^-1^) |
| --- | --- | --- | --- | --- | --- | --- |
| 1639 | 63.6 | 552.9 | 2255.5 | 178.9 | 3.7 | 293.4 |

$$Gravimetric energy density \left( Wh {kg}^{-1} \right)=\frac{Pouch cell capacity \times Working voltage}{Total mass of electrodes}$$

**Table S3.** Calculation details for the volumetric energy density of the bi-layer pouch-type full cell using the IEE system based on electrodes (top) and essential components (bottom).

| Cu foil  (μm) | Anode  (μm) | Al foil  (μm) | Cathode  (μm) | Total electrode thickness  (μm) | Area  (cm^2^) | Capacity  (mAh) | Working voltage  (V) | Volumetric energy density  (Wh L^-1^) |
| --- | --- | --- | --- | --- | --- | --- | --- | --- |
| 19.0 | 34.6 | 15.3 | 100.8 | 169.7 | 30 | 178.9 | 3.7 | 1300.0 |

| Total electrode thickness  (μm) | Separator + electrolyte  (μm) | Total thickness  (μm) | Capacity  (mAh) | Working voltage  (V) | Volumetric energy density  (Wh L^-1^) |
| --- | --- | --- | --- | --- | --- |
| 169.7 | 47 | 216.7 | 178.9 | 3.7 | 1018.1 |

$$Volumetric energy density \left( Wh L^{-1} \right)=\frac{Pouch cell capacity \times Working voltage}{Total thickness of electrodes \times Area}$$

**Table S4.** Comparison of the battery performances using Si-based anodes.

| Anode | Cathode | Anode reversible capacity  (mAh g^-1^) | Gravimetric energy density  (Wh kg^-1^) | Volumetric energy density  (Wh L^-1^) | Cycling performance | Limitations | Ref. |
| --- | --- | --- | --- | --- | --- | --- | --- |
| **SiMP** | **LiNi_0.8_Co_0.1_Mn_0.1_O_2_** | **3606** | **403.7** | **1300.0** | **84.8%@100^th^**  **at 0.2 C** | **-** | **This work** |
| SiMP | LiNi_0.8_Co_0.1_Mn_0.1_O_2_ | 3141.2 | 413 | 1022 | 77.0%@150^th^  at 0.3 C | -High n/p ratio (1.4) | S1 |
| SiMP | LiNi_0.8_Co_0.1_Mn_0.1_O_2_ | ~2200 | - | 1048 | 60%@100^th^  at 0.5 C | -Low specific capacity  -Prelithiation | S2 |
| Submicron-Si + Carbon | LiCoO_2_ | 2738 | 537.0 | 585.1 | 81.9%@200^th^  at 1 A g^-1^ | -Low specific capacity  -Submicron-sized Si | S3 |
| SiOMP | LiNi_0.92_Co_0.55_Mn_0.25_O_2_ | 850 | 382 | 960 | 78.1%@500^th^  at 0.2 C | -Low specific capacity  -Prelithiation | S4 |
| Nanosized Si + Graphite | LiNi_0.6_Co_0.2_Mn_0.2_O_2_ | 973 | 300 | 850 | 55.0%@400^th^  at 0.5 C | -Low specific capacity  -Prelithiation  -Nanosized Si | S5 |
| SiNP + Graphite | LiNi_0.88_Co_00.8_Mn_0.04_O_2_ | 1480 | 318 | 665 | 80.0%@750^th^  at 0.5 C | -Low specific capacity  -Nanosized Si | S6 |
| SiO*_x_* + Graphite | Li_1.14_Ni_0.13_Co_0.13_Mn_0.54_O_2_ | 700 | 420 | 449 | 82.0%@300^th^  at 0.2 C | -Low specific capacity  -Prelithiation | S7 |
| Nanosized Si + Graphite | LiCoO_2_ | 517 | - | 1043 | 92.0%@100^th^  at 0.5 C | -Low specific capacity  -Nanosized Si | S8 |
| Carbon coated SiNP | LiNi_0.45_Co_0.1_Mn_1.45_O_4_ | 1802 | 473.6 | 472.3 | 93.8%@200^th^  at 0.5 C | -Low specific capacity  -Nanosized Si | S9 |
| Columnar Si thin film | LiNi_0.8_Co_0.1_Mn_0.1_O_2_ | 3313 | 183 | 806 | 67.0%@50^th^  at 0.5 C | -High n/p ratio (1.9)  -Nanosized Si | S10 |
| Si-coated graphite | LiNi_0.6_Co_0.2_Mn_0.2_O_2_ | 665 | 333 | 932 | - | -Low specific capacity | S11 |
| SiMP | LiNi_0.8_Co_0.1_Mn_0.1_O_2_ | 3137 | 341 | 840 | 85.0%@200^th^  at 0.1 C | -High n/p ratio (1.9) | S12 |

**Supporting References**

[1] M. Je, H. B. Son, Y. J. Han, H. Jang, S. Kim, D. Kim, J. U. Kang, J. H. Jeong, C. Hwang, G. Song, H. K. Song, T. S. Ha, S. Park, Adv. Sci. **2024**, 11, 2305298.

[2] F. Q. Chen, J. W. Han, D. B. Kong, Y. F. Yuan, J. Xiao, S. C. Wu, D. M. Tang, Y. Q. Deng, W. Lv, J. Lu, F. Y. Kang, Q. H. Yang, Natl Sci Rev **2021**, 8, nwab012.

[3] N. Yang, J. H. Sun, R. Shao, Z. J. Cao, Z. P. Zhang, M. L. Dou, J. Niu, F. Wang, Cell Rep. Phys. Sci. **2022**, 3, 5.

[4] R. S. Fu, J. J. Ji, L. Yun, Y. B. Jiang, J. Zhang, X. F. Zhou, Z. P. Liu, Energy Storage Mater. **2021**, 35, 317.

[5] Q. Y. Li, R. Yi, Y. B. Xu, X. Cao, C. M. Wang, W. Xu, J. G. Zhang, J. Power Sources **2022**, 548, 232063.

[6] J. Moon, H. C. Lee, H. Jung, S. Wakita, S. Cho, J. Yoon, J. Lee, A. Ueda, B. Choi, S. Lee, K. Ito, Y. Kubo, A. C. Lim, J. G. Seo, J. Yoo, S. Lee, Y. Ham, W. Baek, Y. G. Ryu, I. T. Han, Nat. Commun. **2021**, 12, 2714.

[7] J. L. Zhao, Z. P. Shi, Z. L. He, Z. Zhou, F. Q. Li, M. Su, Y. F. Zeng, Q. W. Gu, Y. Li, T. T. Li, B. Qiu, Z. P. Liu, J. Power Sources **2023**, 580, 233393.

[8] M. Ko, S. Chae, J. Ma, N. Kim, H. W. Lee, Y. Cui, J. Cho, Nat. Energy **2016**, 1, 1.

[9] S. Q. Chen, L. F. Shen, P. A. van Aken, J. Maier, Y. Yu, Adv. Mater. **2017**, 29, 1605650.

[10] A. Baasner, F. Reuter, M. Seidel, A. Krause, E. Pflug, P. Härtel, S. Dörfler, T. Abendroth, H. Althues, S. Kaskel, J. Electrochem. Soc. **2020**, 167, 020516.

[11] Y. Son, N. Kim, T. Lee, Y. Lee, J. Ma, S. Chae, J. Sung, H. Cha, Y. Yoo, J. Cho, Adv. Mater. **2020**, 32, 2003286.

[12] A. M. Li, Z. Y. Wang, T. Y. Lee, N. Zhang, T. Y. Li, W. R. Zhang, C. Jayawardana, M. Yeddala, B. L. Lucht, C. S. Wang, Nat. Energy **2024**, 1, 1.
